# Regulon-Specific Control of Transcription Elongation across the Yeast Genome

**DOI:** 10.1371/journal.pgen.1000614

**Published:** 2009-08-21

**Authors:** Vicent Pelechano, Silvia Jimeno-González, Alfonso Rodríguez-Gil, José García-Martínez, José E. Pérez-Ortín, Sebastián Chávez

**Affiliations:** 1 Departamento de Bioquímica y Biología Molecular, Universitat de València, Burjassot, Spain; 2 Departamento de Genética, Universidad de Sevilla, Seville, Spain; 3 Sección de Chips de DNA, Servei Central de Suport a la Investigació, Universitat de València, Burjassot, Spain; Yale University, United States of America

## Abstract

Transcription elongation by RNA polymerase II was often considered an invariant non-regulated process. However, genome-wide studies have shown that transcriptional pausing during elongation is a frequent phenomenon in tightly-regulated metazoan genes. Using a combination of ChIP-on-chip and genomic run-on approaches, we found that the proportion of transcriptionally active RNA polymerase II (active versus total) present throughout the yeast genome is characteristic of some functional gene classes, like those related to ribosomes and mitochondria. This proportion also responds to regulatory stimuli mediated by protein kinase A and, in relation to cytosolic ribosomal-protein genes, it is mediated by the silencing domain of Rap1. We found that this inactive form of RNA polymerase II, which accumulates along the full length of ribosomal protein genes, is phosphorylated in the Ser5 residue of the CTD, but is hypophosphorylated in Ser2. Using the same experimental approach, we show that the *in vivo*–depletion of FACT, a chromatin-related elongation factor, also produces a regulon-specific effect on the expression of the yeast genome. This work demonstrates that the regulation of transcription elongation is a widespread, gene class–dependent phenomenon that also affects housekeeping genes.

## Author Summary

Transcription of DNA–encoded information into RNA is the first step in gene regulation. RNA polymerases initiate transcription at the promoter region and elongate the transcripts traveling throughout the gene until reaching the termination sequences. Classical models of transcriptional regulation were focused on the initiation step, but there is increasing evidence for gene regulation after initiation. We have investigated the importance of elongation in gene regulation using the yeast *Saccharomyces cerevisiae*, one of the main experimental systems in modern biology. By comparing the genomic distribution of RNA polymerase molecules with the actual transcriptional signal across the genome, we have detected that many genes are regulated at the elongation level. We show that yeast cells use this step to modulate the expression of several groups of genes, which have to be simultaneously regulated in a very coordinated manner. Genes encoding essential functions, like those related to protein synthesis and respiration, change their transcriptional activities in response to environmental stimuli, without changing in the same extension the amount of RNA polymerase that is physically associated to them. We also show that this kind of regulation, in spite of taking place during the elongation step, can be mediated by promoter-binding transcription factors.

## Introduction

It is well known that RNA pol II accumulates under repressive conditions on some tightly regulated genes of higher eukaryotes, like human *c-Myc* [1] and *Drosophila hsp70* [2]. RNA pol II pausing is in fact a frequent situation since a significant proportion of metazoan genes exhibits paused polymerases at promoter-proximal sites [3]–[8]. Although pauses and arrests during transcription elongation seem to be also common phenomena further downstream [9].

In yeast, almost 2500 repressed genes show poised RNA pol II in the stationary phase [10] but only a few, like *CYC1* and those encoding NTP-biosynthetic enzymes, display an accumulation of RNA pol II at their 5′ region under repressive conditions in exponential growing cells [11],[12]. For NTP genes, transcription regulation works at the level of initiation through an attenuation mechanism [12],[13]. It is not clear whether the accumulation of RNA pol II at the 5′ end in the other cases responds to a pausing phenomenon. In any case, RNA pol II pausing at promoter-proximal sites is not a frequent phenomenon in exponentially growing yeast [14] which has been proposed to reflect the different chromatin organization of the transcription start sites in yeast compared to metazoa [15].

In the last 20 years, biochemical and genetic analyses have revealed a numerous set of factors playing auxiliary roles in RNA Polymerase II (RNA pol II)-dependent transcription elongation [16]. The textbook view of transcriptional machinery is a uniform set of players that all genes require equally. However, it is already well known that the diversity in core promoter elements throughout the genome reflects certain gene-specific roles of the general transcription factors involved in the pre-initiation complex (PIC) assembly. For instance, yeast TATA box-containing genes are highly regulated and preferentially utilize SAGA rather than TFIID if compared to TATA-less promoters [17]. According to such differences, a TBP regulatory network to explain gene-specific differences in the PIC assembly has been proposed [18].

Similarly, several examples of gene-specific roles of elongation factors have been described. Mutations affecting the integrity of the yeast THO complex, involved in transcription elongation and mRNP biogenesis, decrease the expression levels of long transcription units, but do not significantly influence the mRNA levels of the shorter ones driven by the same promoter [19]–[21]. TFIIS, an elongation factor that is dispensable for the expression of most yeast genes, is absolutely required for the activation of *IMD2* in response to NTP depletion [22]. Mammalian splicing factor SC35 also plays a gene-specific role in transcription elongation since its depletion produces an accumulation of inactive RNA pol II on several, but not all, active transcription units [23].

The transcription of the p53-dependent gene p21 does not require the phosphorylation of the carboxy-terminal domain of RNA pol II (CTD) in the serine residue situated at position 2 (Ser2). This indicates that the requirement of P-TEFb for transcription elongation is also gene-specific [24]. The chromatin factor FACT, involved in chromatin remodeling and reassembly during transcription elongation [25],[26], is also dispensable for the expression of p21 [24]. Likewise, the expression of the yeast *CUP1* gene, which can be transcribed by a mutant version of RNA Pol II lacking the CTD [27], is not affected by FACT depletion [28]. Furthermore by comparing five genes under the control of the same promoter, we have previously shown that FACT is not equally required by all the genes during transcription, and that this differential requirement is related to the chromatin configuration of the transcribed region [28].

In this work, we investigated the distribution of actively elongating and total RNA pol II by means of a new methodological approach that combines genomic run-on (GRO) and ChIP-on-chip. We detected significant gene-specific differences in the proportion of active RNA pol II present in the transcribed regions. The effect of FACT depletion was also differential for some gene functional categories such us those encoding mitochondrial proteins, or housekeeping genes encoding cytosolic ribosomal proteins and factors involved in ribosome biogenesis. We found that the transcription elongation of ribosome-related genes responds to regulatory stimuli mediated by the protein kinase A pathway, and by the Rap1 transcription factor for those genes that encode structural ribosomal proteins. We also found that an inactive form of RNA polymerase II, which is phosphorylated in the Ser5 residue of the CTD but is hypophosphorylated in Ser2, accumulates along the full length of these genes, during standard growing conditions.

## Results

### Ribosomal protein genes are enriched in inactive RNA pol II

**Figure 1:**
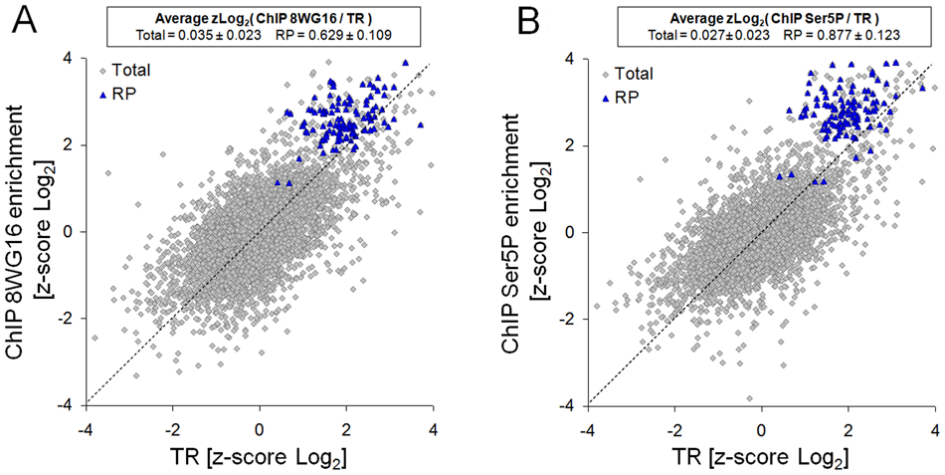
RP genes exhibit higher levels of RNA pol II than expected from their transcription rates. The RP genes of cells exponentially growing in glucose-containing medium are enriched in RNA pol II, as compared to their transcription rates (TR). The levels of RNA pol II were measured by ChIP-on-chip using either the 8WG16 antibody recognizing the RNA pol II CTD (A), or an antibody that specifically recognizes the Ser5-phosphorylated form of Rpb1-CTD (B). The text insert describes the average values for the mean with a confidence interval of 95%, assuming a Gaussian distribution and using the standard error of the sample mean.

We measured the association of RNA pol II with yeast genes in exponentially grown cells in YPD by performing RNA pol II ChIP-on-chip experiments (RPCC, Pelechano et al., to be published elsewhere). All normalized and processed genomic data are included in Table S1. We compared the Rpb1-binding data obtained by RPCC with the transcription rate (TR) data previously measured by GRO [29]. We found that the ribosomal protein genes (RP regulon) were relatively enriched in Rpb1 (using a Myc-tagged version of it, see Figure S1). Even clearer results were obtained when the RPCC experiments were performed with the 8WG16 antibody, which recognizes RNA pol II CTD (Figure 1A). Gene classes relating to cytosolic ribosome and translation presented significantly high ChIP/TR ratios (Table 1). A prominent RNA pol II enrichment was also detected in RP genes with an antibody that recognizes the CTD repeats when these are phosphorylated in the serine residue situated at position 5 (Ser5) (Figure 1B and Table 1). All statistically significant GO categories found in all the genomic experiments are included in Table S2.

**Table 1:** GO categories showing statistically significant enrichment in high or low ChIP/TR ratios, or in TR after Spt16 depletion, in all the genomic experiments done.

Gene Ontology	Experiment									
	*zLog_2_ (ChIP 8WG16/TR)*	*zLog_2_ (ChIP Ser5P/TR)*	*ChIP RNApolII/TR(14.5hYPGal/YPD)*	*ChIP RNApolII/TR (2h YPGal/YPD)*	*ChIP RNApolII/TR (2h YPGal/14.5h YPGal)*	*ChIP RNApolII/TR (Δtpk1/wt)*	*ChIP RNApolII/TR (Δtpk2/wt)*	*ChIP RNApolII/TR(rap1Δsil/RAP1)*	*Log_2_ (TR5h/TRC)*	*Log_2_ (TR7h/TRC)*
structural constituent of ribosome (GO:0003735)	3	6	−36	−22		−5		−7	45	
large ribosomal subunit (GO:0015934)	1	1	−20	−15		−4	−2	−8	23	
translation (GO:0006412)		4	−27	−16		−8	−6	−5	34	
ribosome biogenesis and assembly (GO:0042254)	−2		−43	−13	12	−13	−13		40	3
eukaryotic 48S initiation complex (GO:0016283)	2	6	−21	−5	2	−6	−2	−7	19	
cytosolic part (GO:0044445)	4	8	−39	−22	7	−12	−12	−16	45	1
mitochondrial part (GO:0044429)		−1	15	3	−7	15	10		−8	−3
mitochondrion (GO:0005739)			16	2	−6	9	7		−8	
membrane (GO:0016020)	9	5	9	4	−2	12	14		−17	−4

The data represent the −log10 (adjusted p-value). The positive values represent categories with ratios higher than average, while the negative ones represent those with lower than average ratios. In many cases, blanks represent categories (e.g. ribosome-related) that, although statistically significant when performing a supervised analysis, do not pass the filter of adjusted p-value at 0.05 (FDR test).

We reason that the difference between the GRO and the RPCC data, reflected in the ChIP/TR ratios, could be due to the different degree of accumulation of non-actively elongating RNA pol II either in a step prior to initiation or arrested during elongation (likely back-tracked). Our data indicate that the inactive form of RNA pol II (not producing a run-on signal) which accumulated in RP genes was phosphorylated in the Ser5 residue of the CTD. Therefore, we conclude that it should have passed the initiation step of transcription.

### The accumulation of inactive RNA pol II on ribosome-related genes is regulated in response to metabolic changes

**Figure 2:**
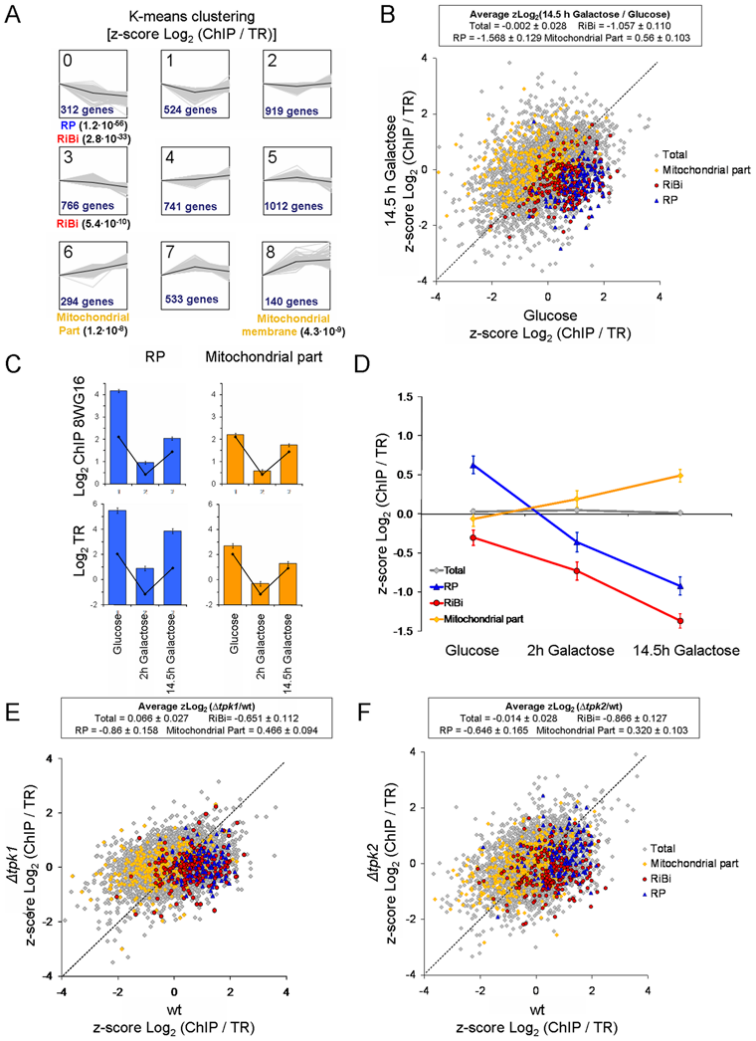
The proportion of active RNA pol II present in RP, RiBi, and mitochondria-related genes is regulated upon the carbon source shift and depends on PKA. (A) K-means clustering analysis of the ChIP/TR profiles of cells exponentially growing in glucose medium (point 1), not growing after shifting to galactose for 2 h (point 2) and exponentially growing in galactose after 14.5 h in this medium (point 3). Graphs represent the 9 clusters (0 to 8) obtained, showing the three-point profiles of the z-scores of the ChIP/TR ratios in log_2_ scale for the group's genes (shaded gray lines) and the average profile line in black. Overrepresented functional gene categories are indicated below together with the p-value of the hypergeometric test of statistical significance. The number of genes present in each cluster is also given. (B) The proportion of active RNA pol II present in both the RP (blue triangles) and the RiBi (red dots) genes is lower in glucose than in galactose. Mitochondria-related genes (orange diamonds) show the opposite pattern. (C) Variation in RNA pol II levels (ChIP with 8WG16) and TRs for RP (blue panels) and mitochondria-related (orange panels) genes under the three conditions described in (A). The error bars show the standard error of the sample mean. Black lines indicate the genome average. (D) Change of ChIP/TR ratios, normalized in relation to the genome average, for the RP, RiBi and mitochondria-related genes during the glucose-galactose shift. The profile shown is equivalent to those shown in part (A) but with the average value for only the functional categories described. The standard error of the sample mean bars are shown. (E, F) The *Δtpk1* and *Δtpk2* mutations mimic the effect of galactose on the ChIP/TR ratios. The proportion of active RNA pol II present is lower for the RP and RiBi genes, and is higher for mitochondria-related genes in *tpk* mutants than in the wild type. In all these experiments, RPCC was performed using the 8WG16 antibody. Text inserts are as in Figure 1. All ChIPs were normalized to a non-transcribed region (chromosome V, intergenic region V).

The detected imbalance between the amounts of RNA pol II bound to RP genes and their TR may either be an intrinsic feature of these genes or reflect the occurrence of a novel mechanism that regulates their expression. In order to test these two possibilities, we calculated the ChIP/TR ratios in three different culture conditions: i) exponential growth in glucose medium, ii) 2 h after transferring glucose-grown cells to galactose-containing medium (non growing cells due to the metabolic shift), and iii) exponential growth in galactose medium (14.5 h in galactose). Then we did a clustering analysis to group genes in accordance with their ChIP/TR patterns. As shown in Figure 2A, two clusters were detected (numbers 0 and 3) in which the ChIP/TR ratio clearly decreased during the shift from glucose to galactose (2 h), and continued to decrease when cells grew exponentially in galactose (14.5 h). The difference between these two clusters was the kinetics of the ChIP/TR decrease, that is, more intense in the first step for cluster number 0 and deeper in the second step for cluster number 3 (Figure 2A). The genes belonging to the RP and RiBi regulons were significantly enriched in cluster 0, although RiBi genes were also located in cluster 3 (Figure 2A). The RiBi regulon comprises all the genes encoding the non RP proteins involved in rDNA transcription, tRNA synthesis, ribosome biogenesis and translation (see [30] for a more precise definition of RiBi). The opposite scenario (higher ChIP/TR ratios in galactose than in glucose) was detected for clusters 6 and 8, which were statistically enriched in mitochondria-related genes. We detected a general genome-wide correlation between the ChIP/TR ratios of cells exponentially growing in glucose and those of cells exponentially growing in galactose, indicating that the lower ChIP/TR ratios shown by the RP and RiBi regulons in galactose and the higher ChIP/TR ratios displayed by mitochondria-related genes indeed reflect a specific regulatory phenomenon (Figure 2B and Table 1).

In the first step of the experiment (2 h), the TR and the amounts of RNA pol II binding to most genes, including the RP regulon, sharply decreased (Figure 2C). This reduction in the genome expression, particularly of the RP genes, is consistent with lack of growth after shifting the culture from glucose to galactose media. Transcription increased in step two (14.5 h) once cells recovered their exponential growth rate, as did the TR of the RP genes and the amount of RNA pol II bound to them (Figure 2C). During these successive down- and up-regulation steps however, the ChIP/TR ratios of RP and RiBi genes continuously decreased in relation to the genome average (Figure 2D; Figure S2A, S2B). This was mainly due to a decrease in the relative amount of bound RNA pol II (Figure S2C) rather than to a relative change in their TR (Figure S2D). Mitochondria-related genes also underwent a similar down- and up-regulation cycle in both their TR and the levels of bound RNA pol II (Figure 2C), although the average ChIP/TR ratios increased in this case (Figure 2D; Figure S2A, S2B). This increase in the ChIP/TR ratio of mitochondria-related genes was also due to a change in the relative amount of bound RNA pol II (Figure S2C) rather than to a variation in the relative TR of respiration genes (Figure S2D). We conclude that the shift from glucose to galactose, and not the growth rate, was the stimulus responsible for the ChIP/TR regulation of RP, RiBi and mitochondria-related genes.

The transcriptional response of RP genes to glucose levels is mediated by the TOR and Ras-PKA pathways [31]. To further confirm that glucose signaling regulates the ChIP/TR ratios of ribosomal related genes, we analyzed a *Δtpk1* mutant. Tpk1 is one of the three PKAs present in yeast and it is physically located on those genes which are highly transcribed [32]. As shown in Figure 2E, the ChIP/TR ratio of RP and RiBi genes lowered, while the mitochondrial genes increased in the *Δtpk1* when compared to an isogenic wild type grown in YPD. The results of *Δtpk1* resembled those of the wild type in galactose (compare Figure 2B and 2E).

Tpk2 is a second PKA catalytic subunit which is physically located on the promoter regions of RP genes [32]. The results of analyzing a *Δtpk2* mutant showed similar patterns to those observed in *Δtpk1*. We did not observe any significant variation in the ChIP/TR ratios of RP genes between *Δtpk1* and *Δtpk2*. We conclude that PKA mediates the signal which regulates the ChIP/TR ratios of RP, RiBi and mitochondrial genes in response to the glucose-galactose shift, but its participation in this regulation does not depend on a particular catalytic subunit.

### Genes encoding ribosomal proteins are enriched in Spt16

**Figure 3:**
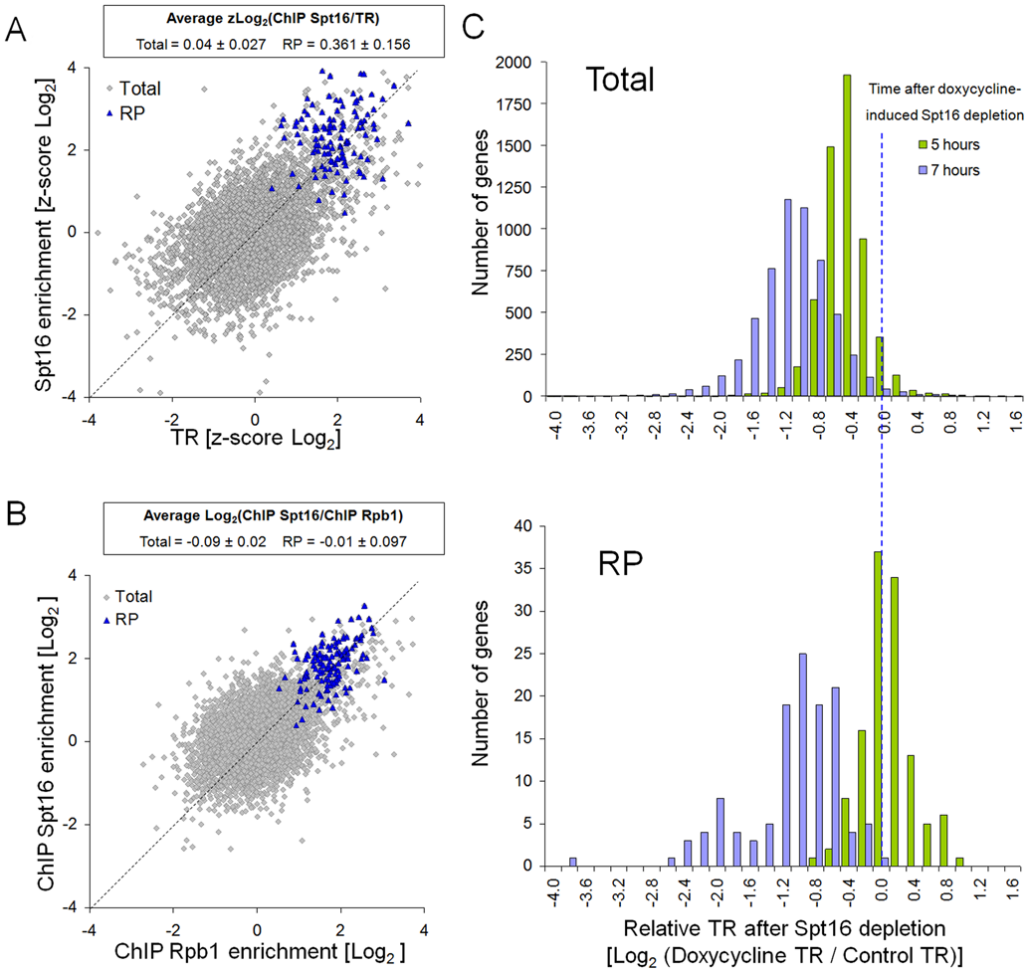
RP genes are enriched in FACT in relation to their transcription rates, but FACT levels correlate with RNA pol II. (A) RP genes present higher Spt16-Myc levels, as measured by ChIP-on-chip, than expected from their TRs. The dashed bisector line indicates the ratio 1. (B) No enrichment of Spt16 on the RP genes, in relation to the Rpb1-Myc levels, was measured by ChIP-on-chip. (C) The transcription rates (TR) of the total (upper panel) and RP (lower panel) genes, measured by genomic run-on, are transiently resistant to Spt16 depletion at 5 h, but not at 7 h. TRs are given as the ratios to the corresponding ones after 5 h of mock treatment. Text inserts are as in Figure 1.

The chromatin elongation factor FACT is formed in yeast by Spt16, Pob3 and Nhp6 [33]. It has been shown that FACT is physically located on active yeast genes along the whole length of their transcription units [34]. In order to test whether the accumulation of inactive RNA pol II on RP genes in glucose is extensive to transcription elongation factors, we measured the amount of Spt16 bound to yeast genes by performing ChIP and by hybridizing the same kind of arrays as we used for GRO and RPCC experiments. As Figure 3A depicts, there is a general positive correlation between the amount of Spt16 bound to a gene and its TR (see also Figure S3A), and similarly to RNA pol II, the RP genes show higher levels of Spt16 than those expected for their TR (Figure 3A and Figure S3B). In contrast, the RP genes showed no Spt16 enrichment in relation to the amount of RNA pol II bound to them (Figure 3B). Irrespectively of the TR, we found a close correlation between RNA Pol II and Spt16 levels of occupancy, as well as a constant Spt16/Rpb1 ratio (Figure S3C), which suggests that the presence of RNA pol II on a gene, rather than its transcriptional activity, causes FACT recruitment.

By using a Tet-off::*SPT16* strain, we have previously shown that there is some gene-specificity in the effect of Spt16 depletion on yeast transcription [28]. In order to test whether the excess FACT present in RP genes was dispensable for their actual transcription rates in glucose, we analyzed the transcriptional effect of Spt16-depletion on a genome-wide scale. Following the GRO procedure, we were able to calculate the effect of Spt16 depletion on TR of 5257 genes, which represents 91% of the genes present in the yeast genome. We took these measurements at depletion times at which neither the growth rate nor the viability of the cells was affected. Five hours after adding doxycycline the overall mRNA levels in the cell were not affected (Figure S4A) but the TR of most genes had decreased (Figure 3C and Figure S4B), which is in agreement with the general positive roles played by FACT in transcription [35],[36].

By carrying out a gene-ontology analysis of the TR decrease, we detected functional classes of genes that were particularly sensitive or insensitive to Spt16 depletion. We found that the RP and RiBi regulons were especially resistant to Spt16 depletion (Figure 3C and Table 1), whereas those genes related to the mitochondria were ranked as hypersensitive (Table 1). Since ribosomal proteins genes are generally short and contain introns, we analyzed the influence of several structural gene features on sensitivity to Spt16 depletion. No correlation with gene length, G+C content or intron presence was found (Figure S5A, S5B, S5C). Since RP genes are highly transcribed, we also checked the influence of TR itself on the response to Spt16 depletion. We found a linear correlation between TR under depletion conditions and control conditions, thus ruling out that highly expressed genes were proportionally less sensitive to Spt16 depletion (Figure S5D).

We performed additional GRO experiments with doxycycline-treated cells over a longer time to achieve a more severe depletion of Spt16. As shown in Figure 3C, the RP regulon showed a similar distribution of TR to the rest of the genome after 7 h of treatment with doxycycline. At this depletion time almost no overrepresentation of gene ontology classes was observed (Table 1). These results confirm that the slight accumulation of FACT on the RP and RiBi regulons, in relation to the levels of actively elongating RNA pol II present, makes these genes transiently resistant to Spt16 depletion. Collectively, these results suggest that not only RNA pol II, but additional elements of the transcription elongation machinery, are enriched on the ribosome-related genes in glucose, if compared to their TR.

### Inactive RNA pol II accumulates in glucose in the body of RP genes

**Figure 4:**
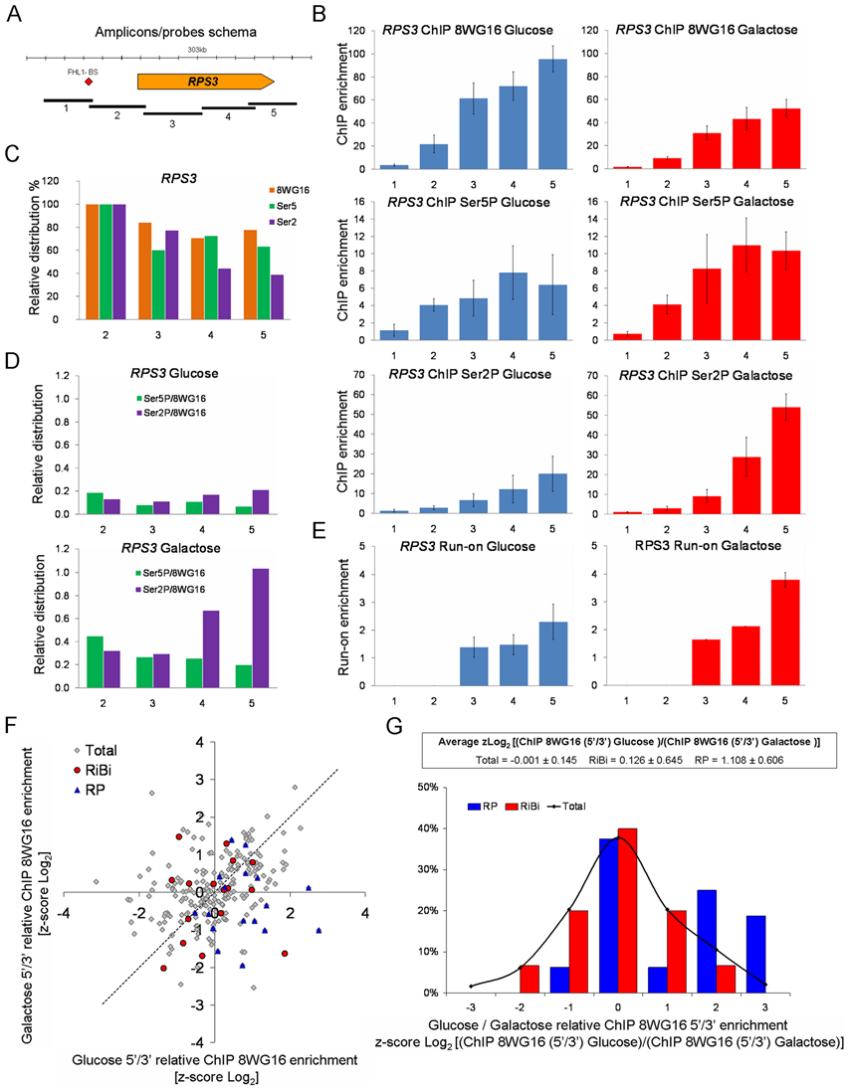
Intragenic distribution of different forms of RNA pol II in RP genes. (A) The amplicons/probes used for RNA pol II ChIP and run-on analyses of *RPS3*. (B) ChIP distribution of total RNA pol II (upper panels) and its phosphorylated CTD forms, in Ser5 (second-line panels) and Ser2 (third-line panels) in cells exponentially growing in glucose (blue bars) or galactose (red bars). The averages of four experiments are shown. Error bars represent the standard deviation. (C) Profile of intragenic RNA pol II distribution in *RPS3* in glucose in relation to its distribution in galactose and to the levels of RNA pol II present in the promoter region. Data are relative to probe 2 because the probe 1 data are very low, producing high error levels in relative calculations (not shown). (D) The relative distribution of phosphorylated forms of RNA pol II CTD with regard to the total amount measured by ChIP. Note the lower relative levels of phosphorylated forms in glucose and the opposing behaviors along the gene of the Ser2 and Ser5 phosphorylated forms in galactose. (E) Run-on distribution in cells exponentially growing in glucose (blue bars) or galactose (red bars). The averages of two experiments are shown. Results were normalized according to the signal of the probes of *PRI2* present on the same filters, as described in Materials and Methods. Error bars represent the standard deviation. (F) The intragenic distribution of RNA pol II in RP genes, measured by ChIP-on-chip using an array of 5′ and 3′ probes of 231 highly expressed genes, is biased toward the 5′ end of the coding region in glucose in relation to the distribution in galactose. (G) Unlike the RP genes, the RiBi genes show almost the same glucose/galactose pattern as the RNA pol II intragenic distribution as the other non RP genes present in the array. Text inserts are as in Figure 1.

The exceeding signal of RPCC over GRO in glucose for RP genes suggests that an accumulation of non-transcribing RNA polymerases takes place. The accumulation of inactive RNA pol II on ribosome-related genes is compatible with a post-recruitment mechanism of transcription regulation. With paused metazoan genes, the intragenic distribution of RNA pol II is biased toward the 5′ end. In order to know whether the RNA pol II enrichment of RP genes also involves a biased distribution of the enzyme, we analyzed in detail the distribution of RNA pol II on a representative RP gene (*RPS3*) by ChIP (Figure 4A–4D). As expected, we found higher levels of total RNA pol II in glucose than in galactose (Figure 4B), but we observed lower levels of Ser5-, and much lower levels of Ser2-phosphorylated RNA pol II in glucose on the 3′ end of the transcribed region (Figure 4B). These different intragenic distributions of RNA pol II are fully compatible with a lower elongation efficiency of RNA pol II in glucose in relation to galactose. The representation of the data following the normalization procedure described by [37] supports this conclusion (Figure 4C). Likewise, the representation of the levels of phosphorylated RNA pol II, normalized by the total levels of the enzyme, reveals a clear difference between the two conditions. Whereas phosphorylation in galactose followed the standard pattern, with a moderate decrease of Ser5-phosphorylation along the gene and a sharp increase of Ser2-phosphorylation towards the 3′ end, the increase of Ser2-phosphorylation in glucose along the gene was considerably less evident (Figure 4D). Very similar results were obtained with the detailed analysis of the gene encoding ribosomal protein L25 (Figure S6A, S6B, S6C, S6D).

We also investigated the intragenic distribution of active RNA pol II by performing a detailed run-on analysis of the *RPS3* gene. In this case, we found similar patterns in both glucose and galactose with comparable levels on the 3′ and medium regions of the transcribed region, and with higher levels at the 3′ end of the gene in galactose than in glucose (Figure 4E). These results support the hypothesis that the accumulation of RNA pol II on RP genes in glucose took place during elongation and was due to a transcriptionally inactive form of RNA pol II that lacked normal levels of Ser2 phosphorylation.

In order to confirm the variation in the intragenic distribution of RNA pol II in RP genes from glucose to galactose, we repeated the RPCC experiments described before with a new type of DNA macroarrays containing 300 bp-long probes covering separately the 5′ and the 3′ ends of the transcribed regions of a set of randomly chosen genes (Rodríguez-Gil et al, submitted). We found that most of the RP genes present in this array presented a higher RPCC 5′/3′ ratio in glucose than in galactose (Figure 4F and 4G). This seems to be specific for RP genes since the RiBi genes represented in the array showed similar RPCC 5′/3′ ratios in the two media, as most non ribosomal genes did (Figure 4F and 4G). We also discovered that neither RP nor RiBi genes showed significantly higher GRO 5′/3′ ratios in glucose than in galactose, thus confirming that the enrichment of RNA pol II located toward the 5′ end of RP genes in glucose consisted of transcriptionally inactive molecules (Figure S6E).

### The silencing domain of Rap1 mediates the post-recruitment regulation of RNA pol II transcription in RP genes

**Figure 5:**
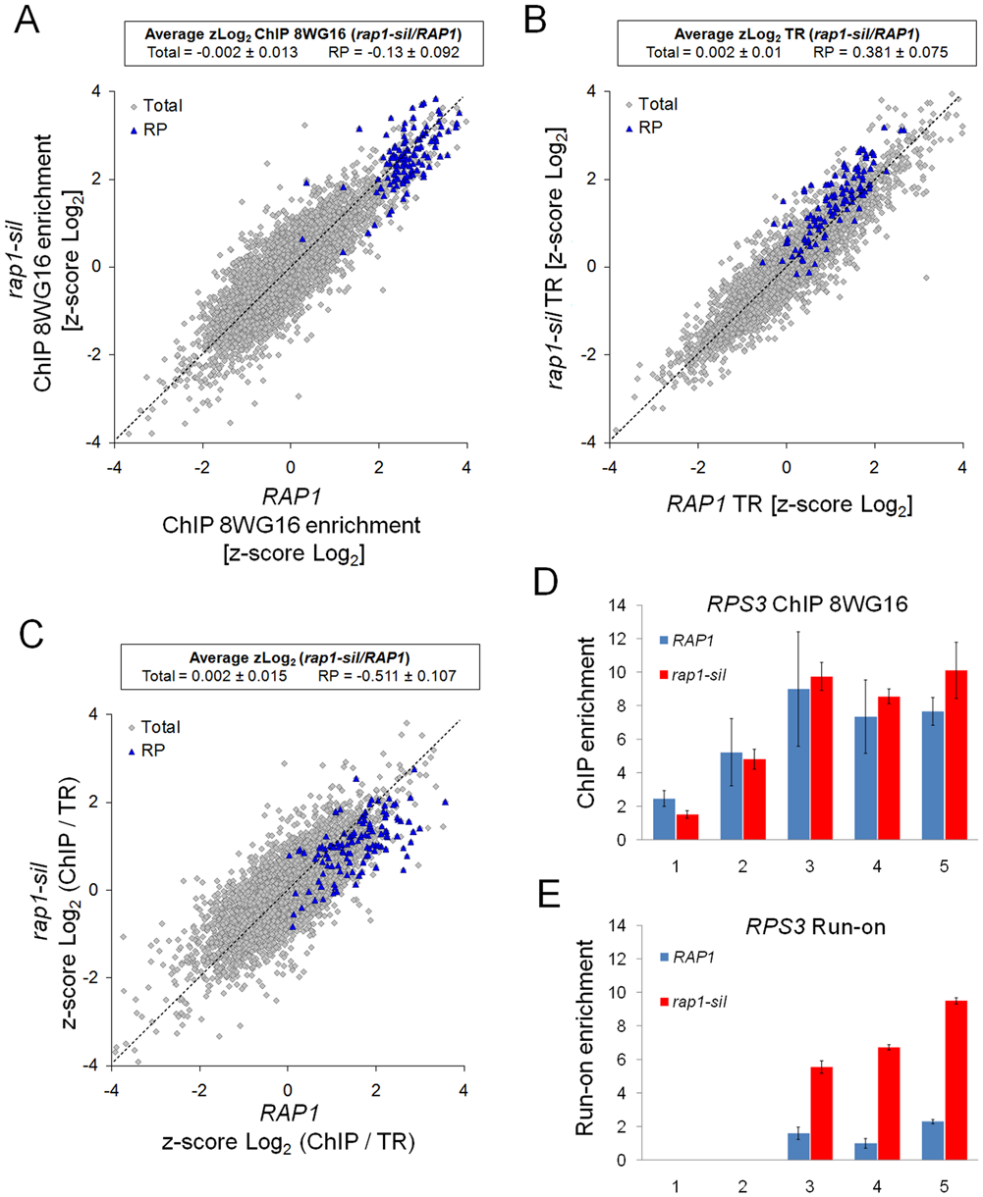
Ablation of the Rap1 silencing domain increases the proportion of active RNA pol II on RP genes. (A) RNA pol II levels, as detected with the 8WG16 antibody, on the RP genes are not significantly affected by the *rap1Δsil* mutation. (B) The *rap1Δsil* mutation increases the levels of active RNA pol II, as detected by run-on, on RP genes. (C) The proportion of active vs. total RNA pol II on the RP genes is significantly lower in the *rap1-Δsil* mutant than in the wild type (*RAP1*). (D) Although there is no significant difference in the distribution of total RNA pol II molecules within the *RPS3* gene, there is a significant increase (E) of the active RNA pol II molecules toward the 3′ end of this gene in the *rap1-Δsil* mutant in relation to the wild type. Run-on data were normalized according to the signal of the probes of *PRI2* present on the same filters, as described in Materials and Methods. The data shown in the histograms correspond to the average of two experiments. Error bars represent standard deviation. Text inserts are as in Figure 1.

So far we have described a novel regulated phenomenon affecting the RP genes expression. It is expected that the mechanism underlying it would be operated by the transcription factors that specifically regulate these genes. A transcription factor playing a mayor role in RP genes transcription is Rap1, a multifunctional protein that also acts as the main duplex DNA binding protein at telomeres, which not only contributes to silencing in both the subtelomeric regions and the mating type loci, but also activates the transcription of glycolytic genes (reviewed by [38],[39]). Rap1 is essential for the RP expression as it organizes chromatin configuration at the RP genes promoters and allows the binding of Fhl1-Ifh1, that is, the other two main transcription factors regulating the transcription of RP genes [40]. An important domain of Rap1 is its silencing domain, which is involved in the subtelomeric recruitment of factors that regulate telomere length and gene silencing [41]. Since mutants lacking the silencing domain of Rap1 are viable and do not show reduced levels of RP gene expression [42], we decided to measure the influence of this mutation on the level of RNA pol II bound to RP genes and on their TR. As shown in Figure 5A, RP were the most enriched genes in RNA pol II in both the wild-type and the *rap1Δsil* mutant. However, and importantly, they were more transcribed in *rap1Δsil* than in the wild type (Figure 5B). Consequently, RP genes displayed a significantly low ChIP/TR ratio in the *rap1Δsil* mutant (Figure 5C). As expected, mitochondria-related genes were unaffected by *rap1Δsil* mutation (Figure S7). The ChIP/TR ratios of RiBi genes, most of which are not directly regulated by Rap1, did not undergo mayor change either (Figure S7C). In this case, they displayed slightly higher levels of both RNA pol II binding and transcriptional activity (Figure S7A, S7B), which probably reflect their upregulation in response to the overexpression of RP genes caused by *rap1Δsil*. As expected, *rap1Δsil* mutation also led to an increase in the TR of subtelomeric genes, but not in RNA pol II binding (Figure S8).

We also investigated the distribution of RNA pol II along *RPS3* in the *rap1Δsil* mutant. We found no clear difference in the intragenic distribution of bound RNA pol II when compared to the wild type (Figure 5D). However, we detected higher levels of active RNA pol II in the mutant measured by run-on, throughout the transcribed region (Figure 5E). Similar results were found for *RPL25* (Figure S9A, S9B). We conclude that the silencing domain of Rap1 participates in the mechanism which controls the proportion of RNA Pol II that is effectively active on RP genes during transcription elongation.

## Discussion

### The proportion of recruited RNA pol II machinery that is transcriptionally active is gene-specific

In this work, we show that the transcriptionally active proportion of RNA pol II bound across the genome is gene-specific and can be regulated in response to physiological stimuli. The presence of glucose causes an accumulation of inactive RNA pol II on RP genes. FACT, a general chromatin factor that is recruited to transcribed genes, also presents an uneven distribution, similar to that shown by RNA pol II. This indicates that not only RNA pol II accumulates on some genes, but other components of the transcriptional machinery that follow this enzyme during elongation also do. Conversely, the presence of galactose, or more likely, the absence of glucose, leads to a decrease in the proportion of inactive RNA pol II on RP and RiBi regulons, and increases it on mitochondria-related genes. Briefly, a set of at least 1000 genes (more than 15% of the yeast genome) coordinately changes the fraction of RNA pol II that is effectively active during their transcription.

Genome-wide analysis has shown that TOR and PKA pathways co-regulate several gene regulons in yeast, including RiBi, RP and respiration-related genes [31]. Whereas TOR acts as an activator of all three regulons, the PKA pathway acts by repressing respiratory genes and by activating the RP and RiBi genes. Here we show that the absence of either Tpk1 or Tpk2, two of the yeast PKA variants, produces the same kind of changes in the ChIP/TR ratios on RP, RiBi and mitochondria-related genes as the changing growth of the wild type from glucose to galactose. This indicates that the overabundance of inactive RNA polymerases is characteristic of some specific groups of genes, under particular growth conditions, and that it is regulated by the PKA pathway. The fact that there is no difference between the lack of Tpk2, a PKA subunit shown to be bound to RP genes promoters [32] and Tpk1, a subunit bound to the body of most genes suggests that the effect is quantitative: a reduction in PKA activity caused either by the lack of the alternative subunits or the growth in galactose reduces the accumulation of inactive RNA pol II molecules on several kinds of yeast genes.

The importance of gene-specific regulation during elongation across metazoan genomes can be deduced from the occurrence of RNA pol II pausing, which is a frequent phenomenon mainly affecting tightly regulated genes [3]–[7]. Our work indicates that the control of RNA pol II elongation is also a common regulatory mechanism in yeast. Unlike metazoan genes however, whose paused RNA pol II concentrate at specific promoter-proximal sites, elongation-regulated yeast genes, at least *RPS3* and *RPL25*, accumulate inactive RNA pol II along the length of their bodies with only some bias toward their 5′ moiety. This accumulation correlates with a decrease in Ser2-phosphorylated RNA pol II along these genes.

### Transcription of ribosomal genes is regulated during elongation

The experimental evidence described in this work reveals that an excess of RNA pol II, phosphorylated in Ser5, accumulates on the yeast RP genes in glucose. This situation is only compatible with a post-initiation form of RNA pol II. However, the absence of a comparably high run-on signal indicates that this extra amount of RNA pol II, which accumulates in glucose media, should be arrested after backtracking. A similar situation occurs in the Drosophila *hsp70* gene upon depleting the TFIIS cleavage factor [43].

Regulation of ribosome synthesis is a key element in controlling cell homeostasis, cell size and proliferation [30]. A coordinated and balanced expression of all the ribosomal protein genes is also needed to ensure efficient ribosome assembly [44], and to avoid the potential toxicity of free ribosomal proteins [45]. Regulation at the transcription elongation level may provide a gear box-like mechanism which enables a fine-tuning of RP and RiBi transcription by rapidly adjusting the proportion of recruited machinery that is effectively active in response to the specific translational requirements of each physiological state. According to a recently proposed model, a certain level of backtracking during elongation, in combination with a fast initiation step, provides a steadier mRNA population level than that which would be produced by an initiation model alone [46]. Accordingly, the regulation of RP transcription elongation would allow the expression of balanced amounts of translational machinery components. It would also contribute to avoid transcription bursts, which would be incompatible with the low transcriptional noise that characterizes yeast constitutive genes [47] and, more specifically, the RP expression [48]. In addition, and as suggested for *Drosophila* genes [5], regulating the transcription at the elongation level enables a continuously open promoter configuration, an essential situation for genes like RP which are being permanently transcribed.

A feedback regulation mechanism operating at the intron splicing level has been demonstrated for certain RP genes [49]. Since exon definition takes place during transcription elongation, an attractive hypothesis would be the existence of coordination between transcription elongation and RNA splicing in RP genes [50]. However, our data do not support such a hypothesis since RNA pol II enrichment, compared to TR, was detected in both intron-containing and intron-less RP genes (data not shown).

RP and RiBi regulons show different RNA pol II- and FACT-ChIP/TR ratios, which suggest that their regulation mechanisms are not identical. We have also detected this kind of regulation in the group of mitochondria-related genes, thus confirming the previously described control of *CYC1* transcription after RNA pol II recruitment [11],[51]. In this case, the ChIP/TR ratios were reciprocal to those of RP genes. If we consider this diversity, it is likely that at least one subset of the molecular elements regulating the proportion of active RNA pol II during elongation is gene-specific.

### Rap1 regulates transcription elongation

**Figure 6:**
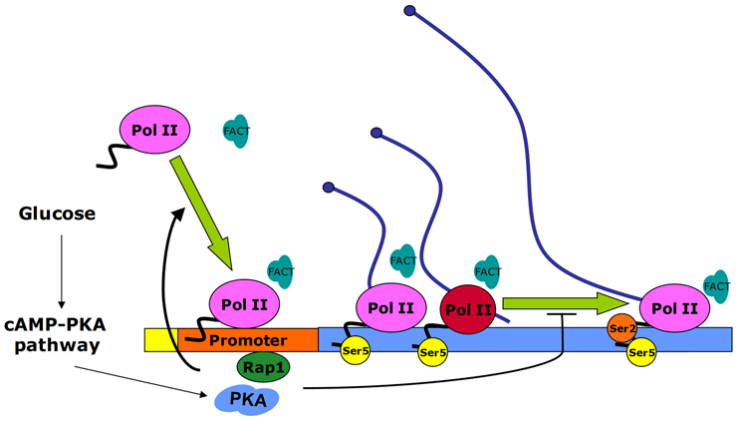
Model for the regulation of transcription elongation in RP genes. Glucose stimulates RNA pol II recruitment to the RP genes by means of the cAMP-PKA pathway. FACT would be recruited to the RP genes together with RNA pol II. PKA would also inhibit transcription elongation. These two actions would be mediated by the DNA-binding RP-specific transcription factor Rap1, whose silencing domain is responsible for the elongation-inhibitory function. Inhibition of elongation would favor RNA pol II backtracking, leading to an increase in the proportion of inactive polymerases on the body of RP genes. RNA pol II molecules are shown in different colors and shapes according to their activity state: initiating or actively elongating (pink) and backtracked (red).

We provide evidence for the specific involvement of the silencing domain of Rap1 in the mechanism required to maintain significant levels of inactive RNA Pol II on RP genes. We also show that the absence of either Tpk1 or Tpk2 produces the same phenotype on RP transcription. These results indicate that the proportion of inactive RNA pol II on RP genes is controlled by the factors that specifically regulate the transcription of RP genes. In such a scenario, Rap1 would regulate the transcription of RP genes at both the RNA pol II recruitment and transcription elongation levels (Figure 6).

Tethering experiments using lexA-Rap1 chimeras have shown that the Rap1 DNA-binding domain itself is required for the activating function of Rap1 in RP transcription [40]. This observation, together with the ability of Rap1 to clear nucleosomes from the vicinity of its binding sites [52], suggests that the positive contribution of Rap1 to RP transcription is exerted in a pre-initiation step. This is likely to be done by arranging a chromatin configuration of the promoter to allow the hosting of other RP transactivators like Fhl1-Ifh1 [40] and, eventually, the pre-initiation complex itself. The persistent occupancy of Rap1 on RP promoters, even under transcriptionally inactive conditions (stress), suggests that this factor may also play a repressive role [53]. We provide evidence of a negative role of Rap1 on RP transcription elongation which is mediated by its silencing domain. This domain, located in the C-terminal part of the protein, has been previously shown to be important for the downregulation of RP transcription in response to certain defects in the secretory pathway [54],[55]. Graham et al. [42] showed that it also affects the mRNA steady-state levels of RP genes. They attributed this effect to the secondary post-transcriptional consequences of the *rap1Δsil* deletions. Our results indicate that it is in fact a transcriptional effect since the silencing domain has a negative influence on the TR of both subtelomeric (Figure S8) and RP genes (Figure 5) without affecting RNA pol II recruitment.

In *Drosophila* cells, hundreds of genes show that RNA polymerase II molecules paused after initiation (about 20–50 bp from the TSS), which has been argued to deal with the presence of an H2AZ-containing positioned nucleosome [56]. In yeast, the advanced position of the first nucleosome, overlapping the TSS [15], makes such a mechanism unlikely. However and as we show herein, the regulation of the chromatin configuration by DNA-binding proteins like Rap1 may also have an effect on elongation.

The Rap1-dependent control that we propose for RP genes should not be the only one acting at the elongation level across the yeast genome as we have shown that at least two other functional groups of genes, RiBi and mitochondria-related genes, display a regulated variation in the proportion of active RNA polymerases. This variation is controlled by PKA, but does not depend on the silencing domain of Rap1. It is tentative to hypothesize that the PKA pathway regulates a plethora of genes during the elongation step of transcription by using different chromatin-related factors.

## Materials and Methods

### Yeast strains, growth conditions, and FACT depletion

The yeast strains used in this work are described in Table S3. Cells were grown in YPD (yeast extract 1%, peptone 2%, glucose 2%) with agitation at 28°C, at OD_600_ = 0.5. In the experiment done in the galactose medium, cells were harvested and changed to YPGal (yeast extract 1%, peptone 2%, galactose 2%) and grown for 2 h (lag phase) and 14.5 h (exponential growth).

For the *SPT16* shut-off experiments, 5 µg/ml doxycycline was added to exponentially growing SJY6 cells (OD_600_ = 0.1). Since the experiments in this work were performed in rich media (YPD), shorter times of incubation with doxycycline were required to reach the same level of Spt16 depletion described in [28]. Control cells were harvested after 5 hours of mock treatment.

### Genomic run-on

Genomic run-on (GRO) was done essentially as described in [29]. See supplementary materials and methods in Text S1.

### Small-scale run-on for selected genes

To determine the intragenic distribution of elongating RNA pol II molecules, we used macroarrays containing 300 bp probes from the 5′ and 3′ ends of the coding regions of 377 yeast genes. These 5′-3′ macroarrays were manufactured by printing PCR products onto a nylon Hybond N+ membrane, similarly to that described for whole genome ORF macroarrays [57]. PCR products were obtained by using either yeast genomic DNA as a template and the primer pairs listed in Table S4, or a plasmid containing the ORF, a common primer corresponding to the plasmid (YGUF for 5′ probes or YGUR for 3′ probes), and a specific primer corresponding to the ORF, listed in Table S5, following the procedure described in [57].

The run-on analysis of *RPS3* and *RPL25* was done as in GRO experiments, but using miniarrays on nylon Hybond N+ membranes. These miniarrays were done by printing the PCR products as described above using the amplicons listed in Table S6. Probes of *PRI2*, a gene whose run-on levels were not influenced by any of the elements tested in the GRO experiments, were included for normalization.

### Chromatin immunoprecipitation of RNA pol II and RPCC

A detailed protocol of RPCC will be published elsewhere (Pelechano and Pérez-Ortín, submitted). A preliminary description of it is included in the supplementary materials and methods (Text S1).

ChIP experiments of selected genes were performed as previously described [28] with minor modifications. Shortly, 50 ml of yeast culture were collected at O.D. 0.5. Crosslinking was performed by adding 1% Formaldehyde to the culture and incubating at room temperature for 15 min. 2.5 ml of glycine was then added and culture was incubated 5 min. Cells were then harvested and washed four times with 25 ml Tris-HCl Buffer Saline at 4°C. The cell breakage was performed in 300 µl of lysis buffer (see the above reference) with glass beads, and the cell extracts were sonicated in a Bioruptor sonicator (Diagenode) for 30 min in 30 sec on/30 sec off cycles (chromatin was sheared into an average size of 300 bp). Immunoprecipitation was performed with magnetic beads, which were coated with protein A (Dynal) and incubated with 8WG16 monoclonal antibody (Bavco Covance), anti Ser2-P-CTD or anti-Ser5-P-CTD (kindly provided by David Bentley) beforehand.

qPCR were performed to quantify immunoprecitation, using a 1∶1500 dilution for the input samples or a 1∶10 dilution for the immunoprecipitated samples. Immunoprecipitation was defined as the ratio of each probe specific product in relation to that of a non-transcribed region (chromosome V, intergenic region V). Primers used are listed in Table S6.

### *In silico* functional analyses

All the experiments were done in triplicate except for the *tpk2* mutant that was analyzed in duplicate. All the group functional enrichment analyses were done using the Fatiscan application from Babelomics [58]. The clustering of Figure 2A was done using a k-means algorithm and the STEM program [59].

### Accession numbers

Genomic data are stored in the Valencia Yeast (VYdBase; http://vydbase.uv.es/) and GEO databases. The GEO accession number for the set of hybridizations is GSE14084.

## Supporting Information

Figure S1 RP genes are enriched in Rpb1 relative to their TR. (A) Rpb1-Myc levels, measured by ChIP-on-chip using an anti-Myc antibody, correlate with TR, measured by GRO. The RP genes are enriched in Rpb1-Myc, in relation to their TR. (B) The RP ChIP/TR ratios distribution (blue bars) is displaced toward higher values compared to the overall genome distribution (Gaussian line). Other symbols and the text insert are as in Figure 1.
(0.46 MB TIF)

Figure S2 RP, RiBi, and respiratory genes show specific changes in the levels of RNA pol II present and in the proportion of active RNA pol II, upon the carbon source shift. The proportion of RNA pol II that is active on RP and RiBi, reflected inversely by the ChIP/TR ratio, increases when cells are shifted from glucose (YPD) to galactose medium (YPGal) for two hours (A) and continues increasing when cultivated further in galactose medium for 14.5 hours (B). Mitochondria-related genes show the opposite pattern. The relative levels of RNA pol II on RP and RiBi genes are lower in galactose than in glucose, whereas they are higher in glucose than in galactose for mitochondria-related genes (C). The relative distribution of all three groups of genes with regard to the overall population in TR values do not change when comparing cells exponentially growing in glucose and in galactose (D). Symbols are as in Figure 1 and Figure 2.
(1.50 MB TIF)

Figure S3 Correspondence between the presence of Spt16 and Rpb1 and the observed TR. (A) Spt16-Myc and Rpb1-Myc, measured by ChIP-on-chip, show a similar correlation with TR as measured by GRO. A smoothness of the data, using 100 genes sliding windows, is represented. As expected, NA (no antibody) does not correlate with TR. (B) The distribution of the Spt16-ChIP/TR ratios for the RP genes (blue bars) is displaced toward higher values, in relation to the overall genome distribution (Gaussian line). (C) The Spt16/Rpb1 ratio does not depend on the transcription rate (TR). The RP genes (blue triangles) show the same average Spt16/Rpb1 ratio as the rest of the genome.
(0.80 MB TIF)

Figure S4 Effects of FACT (Spt16) depletion on yeast transcription. (A) Overall levels of mRNA amounts and TRs during Spt16 depletion. mRNA amounts were calculated as poly(A) per cell, while TR is the total of the GRO signals corresponding to the RNA pol II-dependent genes present in the arrays, as described in M&M. Both were normalized to time 0. (B) Overall distribution of TRs before and after Spt16 depletion.
(0.46 MB TIF)

Figure S5 The changes in TR upon Spt16 depletion do not correlate with the ORF length, G+C content, intron presence or absolute TR. (A) No indication of the dependence of the TR decrease on the gene length after 5 or 7 hours of depletion was observed. Individual genes are shown as gray dots and the tendency line for the sliding mean is shown as a red line. (B) G+C content does not influence TR sensitivity to Spt16 depletion. (C) The presence of introns does not preclude the sensitivity of TR to Spt16 depletion. Among intron-containing genes, only RP show resistance to Spt16 depletion at 5 h after doxycycline addition. (D) After 5 or 7 h of Spt16 depletion, the linear relationship with the control TR shows that absolute TR has no influence on the transcriptional effect of Spt16 depletion.
(1.03 MB TIF)

Figure S6 Intragenic distribution of different forms of RNA pol II in *RPL25* gene. (A) Amplicons/probes used for RNA pol II ChIP and run-on analyses of *RPL25*. (B) ChIP distribution of total RNA pol II (upper panels) and its phosphorylated CTD forms, in Ser5 (second line panels) and Ser2 (third line panels) in cells exponentially growing in glucose (blue bars) or galactose (red bars). (C) Profile of intragenic RNA pol II distribution in *RPL25* in glucose, in relation to its distribution in galactose and to the levels of RNA pol II present in the promoter region. (D) Relative distribution of phosphorylated forms of RNA pol II CTD relative to the total amount measured by ChIP. (E) The intragenic distribution of RNA pol II in RP genes, measured by run-on using an array of 5′ and 3′ probes, is not biased toward the 5′ end of the coding region in glucose, relative to the distribution in galactose. Symbols as in Figure 4.
(0.48 MB TIF)

Figure S7 Effect of *rap1Δsil* on the presence of RNA pol II in the RiBi regulon and mitochondria-related genes. The *rap1Δsil* mutation slightly increases the RNA pol II levels (A) and transcription rates (B) in the RiBi genes (red dots) without affecting their ChIP/TR ratios (C). No effect on either the RNA pol II levels or transcription rates was detected in mitochondria-related genes (orange diamonds).
(1.17 MB TIF)

Figure S8 The *rap1Δsil* mutation increases the proportion of active RNA pol II in subtelomeric genes. The *rap1Δsil* mutation does not produce significant changes along the length of the chromosome at the total RNA pol II levels, measured by ChIP-on-chip (A, B). It does not produce an increase of active RNA pol molecules along length of the the chromosome length either (C), except within the 10 kb region near the telomeres (extended resolution in D). The red and blue lines represent the averages of the values using a sliding window of 50 genes. Horizontal lines represent the upper and lower limit for this mean using a Shewhart chart with a confidence range of 0.999.
(1.22 MB TIF)

Figure S9 Effect of *rap1Δsil* on the presence and activity of RNA pol II in RPL25. There is no significant difference in the distribution of RNA pol II molecules within the RPL25 gene (A), but there is a significant increase of active RNA pol II molecules toward the 3′ end of the gene in the rap1Δsil mutant with regard to the wild type RAP1 (B).
(0.10 MB TIF)

Table S1 Excel file containing all the normalized and processed data.
(5.92 MB XLS)

Table S2 Excel file containing the statistically significant GO categories found in all the genomic experiments.
(3.49 MB XLS)

Table S3 Yeast strains used in this work.
(0.04 MB DOC)

Table S4 Oligonucleotides used for the PCR amplification of the first set of probes printed on the 5′/3′ arrays XLS.
(0.11 MB XLS)

Table S5 Oligonucleotides used for the PCR amplification of the second set of probes printed on the 5′/3′ arrays.
(0.05 MB XLS)

Table S6 Oligonucleotides used for PCR amplification during the detailed studies of *RPL3* and *RPL25*.
(0.01 MB XLS)

Text S1 Supplementary Materials and Methods.
(0.04 MB DOC)
