# Supplementary figures and images for: Regulon-Specific Control of Transcription Elongation across the Yeast Genome
Source: PLoS Genet. 2009 Aug 21;5(8):e1000614. doi: 10.1371/journal.pgen.1000614 (PMC2721418; doi:10.1371/journal.pgen.1000614)

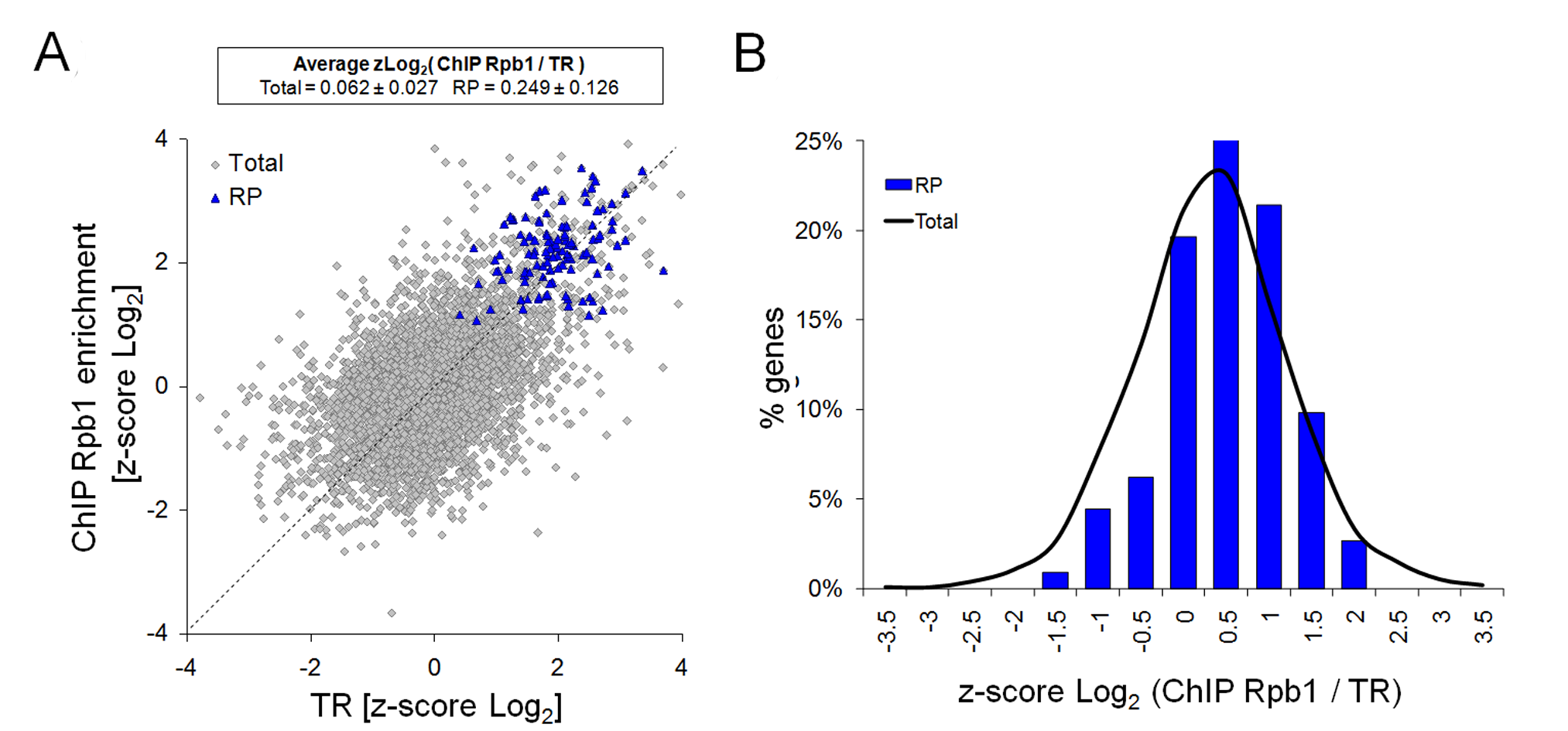

Supplement: Figure S1 — RP genes are enriched in Rpb1 relative to their TR. (A) Rpb1-Myc levels, measured by ChIP-on-chip using an anti-Myc antibody, correlate with TR, measured by GRO. The RP genes are enriched in Rpb1-Myc, in relation to their TR. (B) The RP ChIP/TR ratios distribution (blue bars) is displaced toward higher values compared to the overall genome distribution (Gaussian line). Other symbols and the text insert are as in Figure 1. (0.46 MB TIF) [file pgen.1000614.s001.tif]

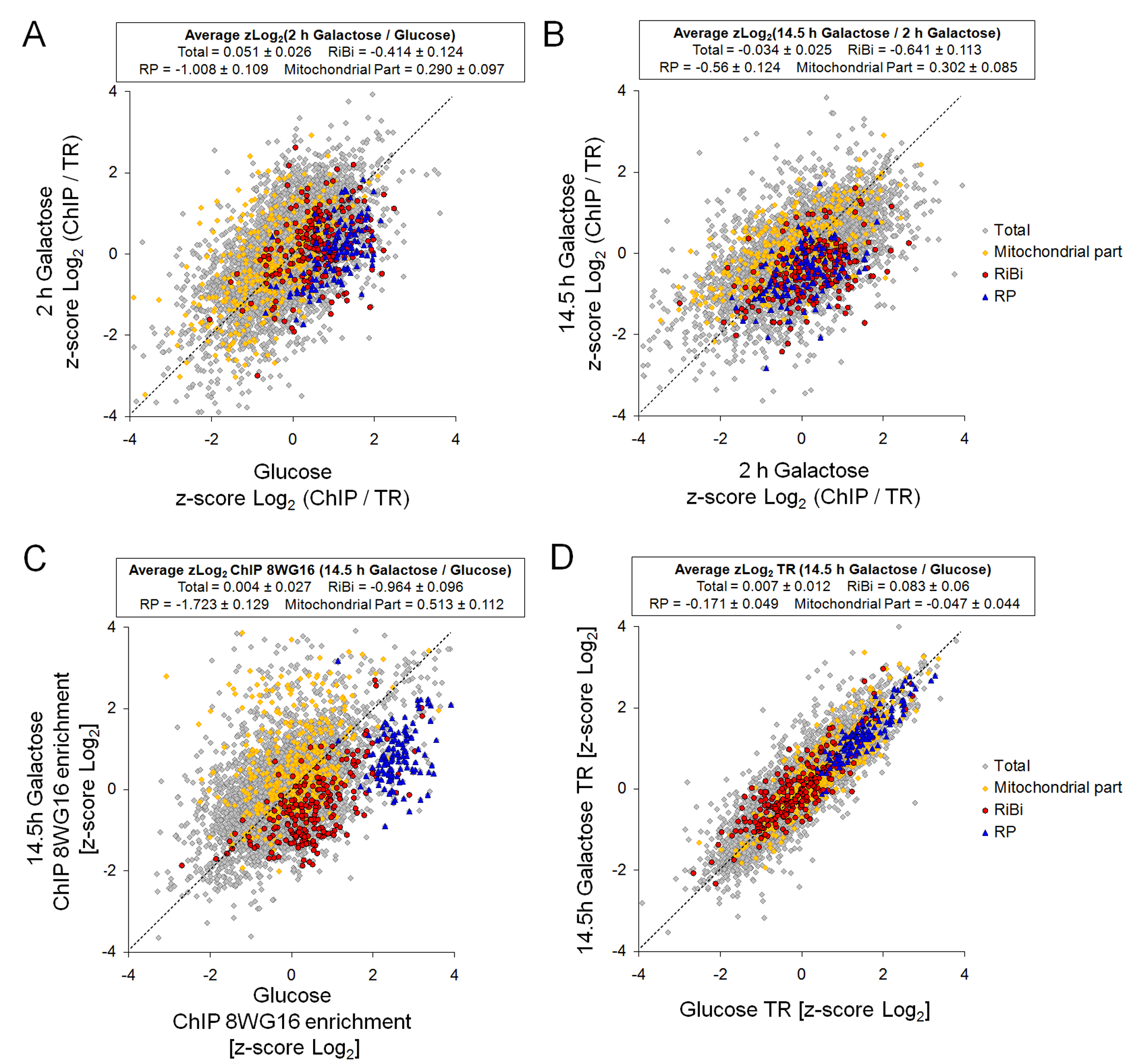

Supplement: Figure S2 — RP, RiBi, and respiratory genes show specific changes in the levels of RNA pol II present and in the proportion of active RNA pol II, upon the carbon source shift. The proportion of RNA pol II that is active on RP and RiBi, reflected inversely by the ChIP/TR ratio, increases when cells are shifted from glucose (YPD) to galactose medium (YPGal) for two hours (A) and continues increasing when cultivated further in galactose medium for 14.5 hours (B). Mitochondria-related genes show the opposite pattern. The relative levels of RNA pol II on RP and RiBi genes are lower in galactose than in glucose, whereas they are higher in glucose than in galactose for mitochondria-related genes (C). The relative distribution of all three groups of genes with regard to the overall population in TR values do not change when comparing cells exponentially growing in glucose and in galactose (D). Symbols are as in Figure 1 and Figure 2. (1.50 MB TIF) [file pgen.1000614.s002.tif]

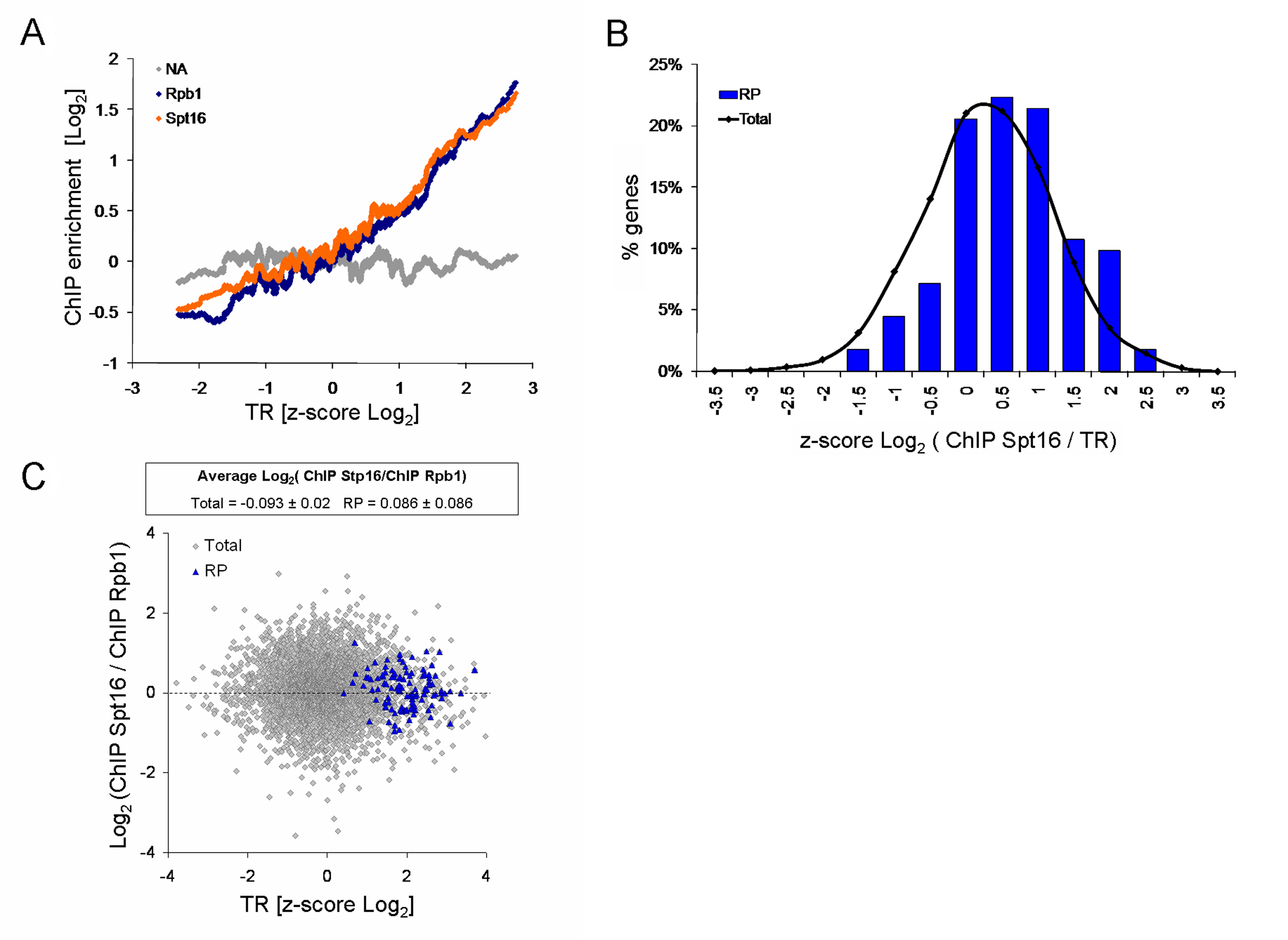

Supplement: Figure S3 — Correspondence between the presence of Spt16 and Rpb1 and the observed TR. (A) Spt16-Myc and Rpb1-Myc, measured by ChIP-on-chip, show a similar correlation with TR as measured by GRO. A smoothness of the data, using 100 genes sliding windows, is represented. As expected, NA (no antibody) does not correlate with TR. (B) The distribution of the Spt16-ChIP/TR ratios for the RP genes (blue bars) is displaced toward higher values, in relation to the overall genome distribution (Gaussian line). (C) The Spt16/Rpb1 ratio does not depend on the transcription rate (TR). The RP genes (blue triangles) show the same average Spt16/Rpb1 ratio as the rest of the genome. (0.80 MB TIF) [file pgen.1000614.s003.tif]

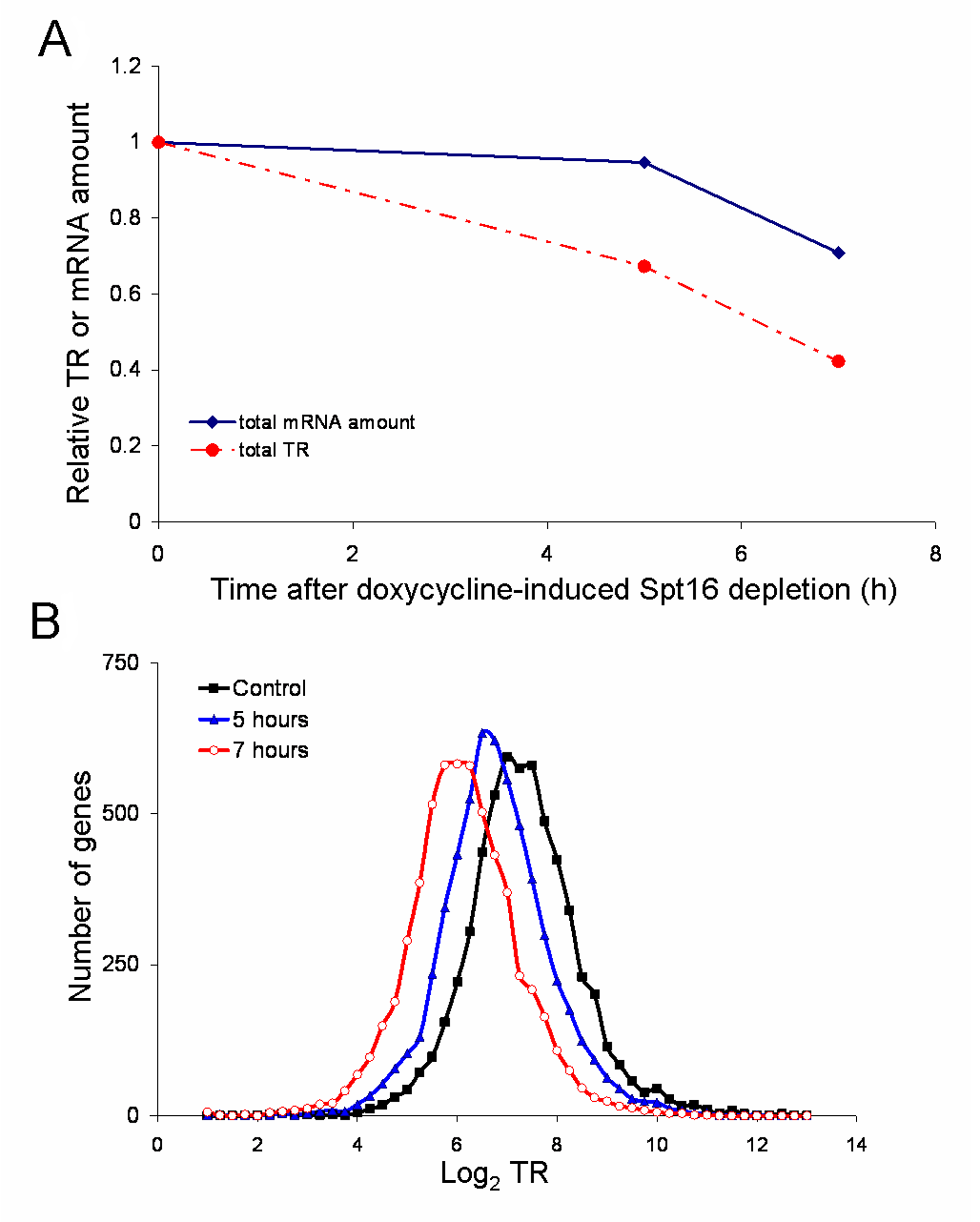

Supplement: Figure S4 — Effects of FACT (Spt16) depletion on yeast transcription. (A) Overall levels of mRNA amounts and TRs during Spt16 depletion. mRNA amounts were calculated as poly(A) per cell, while TR is the total of the GRO signals corresponding to the RNA pol II-dependent genes present in the arrays, as described in M&M. Both were normalized to time 0. (B) Overall distribution of TRs before and after Spt16 depletion. (0.46 MB TIF) [file pgen.1000614.s004.tif]

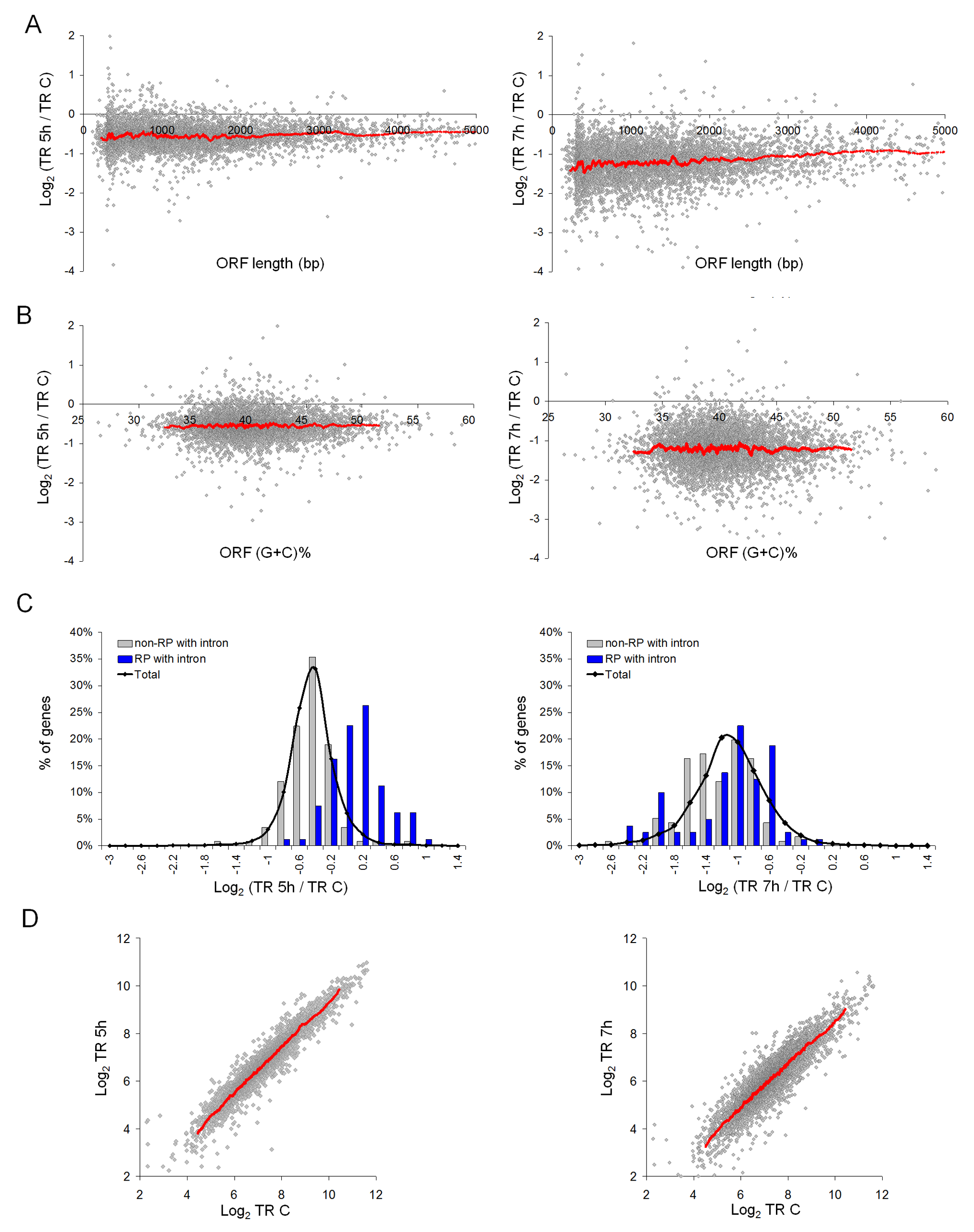

Supplement: Figure S5 — The changes in TR upon Spt16 depletion do not correlate with the ORF length, G+C content, intron presence or absolute TR. (A) No indication of the dependence of the TR decrease on the gene length after 5 or 7 hours of depletion was observed. Individual genes are shown as gray dots and the tendency line for the sliding mean is shown as a red line. (B) G+C content does not influence TR sensitivity to Spt16 depletion. (C) The presence of introns does not preclude the sensitivity of TR to Spt16 depletion. Among intron-containing genes, only RP show resistance to Spt16 depletion at 5 h after doxycycline addition. (D) After 5 or 7 h of Spt16 depletion, the linear relationship with the control TR shows that absolute TR has no influence on the transcriptional effect of Spt16 depletion. (1.03 MB TIF) [file pgen.1000614.s005.tif]

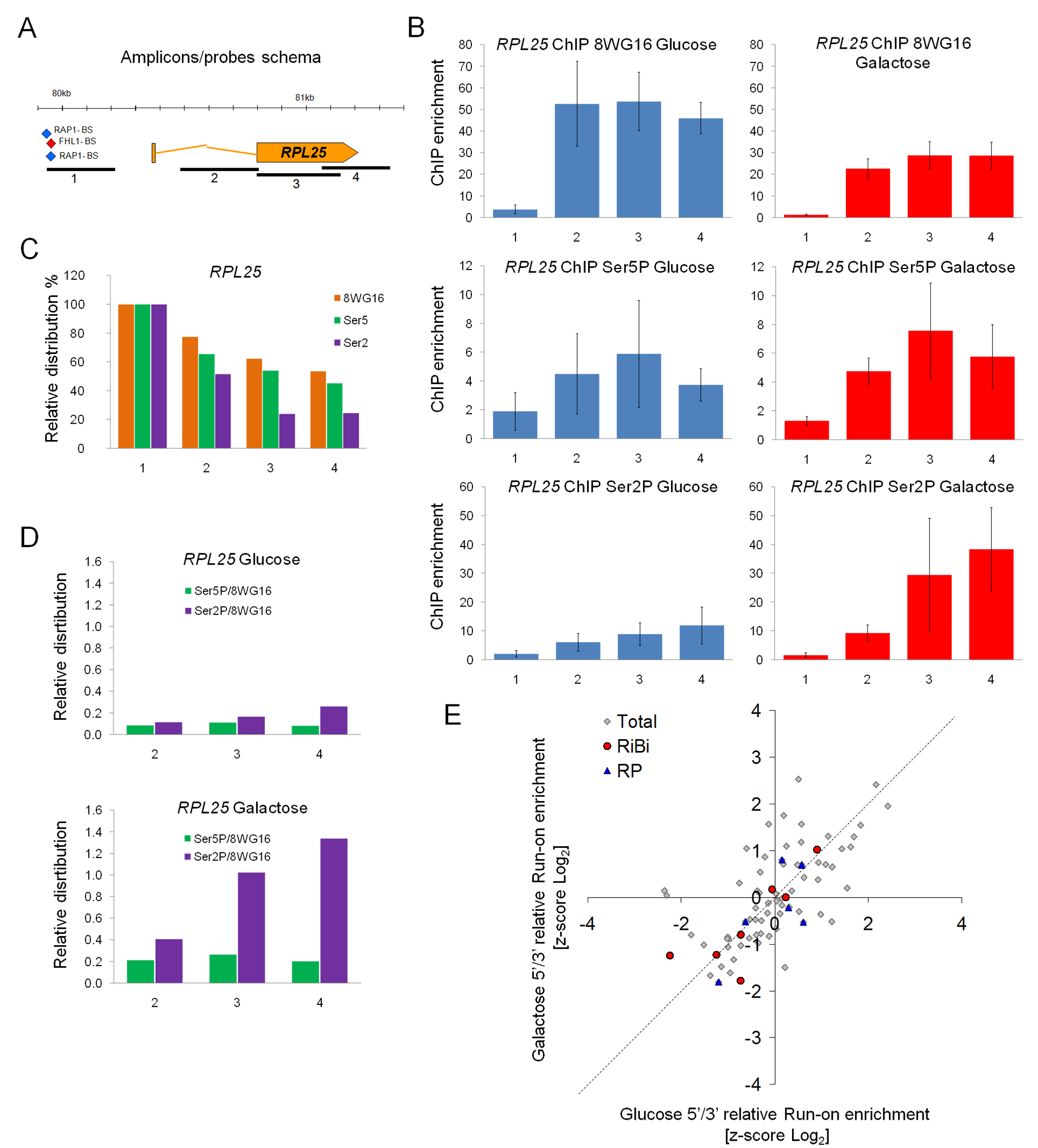

Supplement: Figure S6 — Intragenic distribution of different forms of RNA pol II in RPL25 gene. (A) Amplicons/probes used for RNA pol II ChIP and run-on analyses of RPL25. (B) ChIP distribution of total RNA pol II (upper panels) and its phosphorylated CTD forms, in Ser5 (second line panels) and Ser2 (third line panels) in cells exponentially growing in glucose (blue bars) or galactose (red bars). (C) Profile of intragenic RNA pol II distribution in RPL25 in glucose, in relation to its distribution in galactose and to the levels of RNA pol II present in the promoter region. (D) Relative distribution of phosphorylated forms of RNA pol II CTD relative to the total amount measured by ChIP. (E) The intragenic distribution of RNA pol II in RP genes, measured by run-on using an array of 5′ and 3′ probes, is not biased toward the 5′ end of the coding region in glucose, relative to the distribution in galactose. Symbols as in Figure 4. (0.48 MB TIF) [file pgen.1000614.s006.tif]

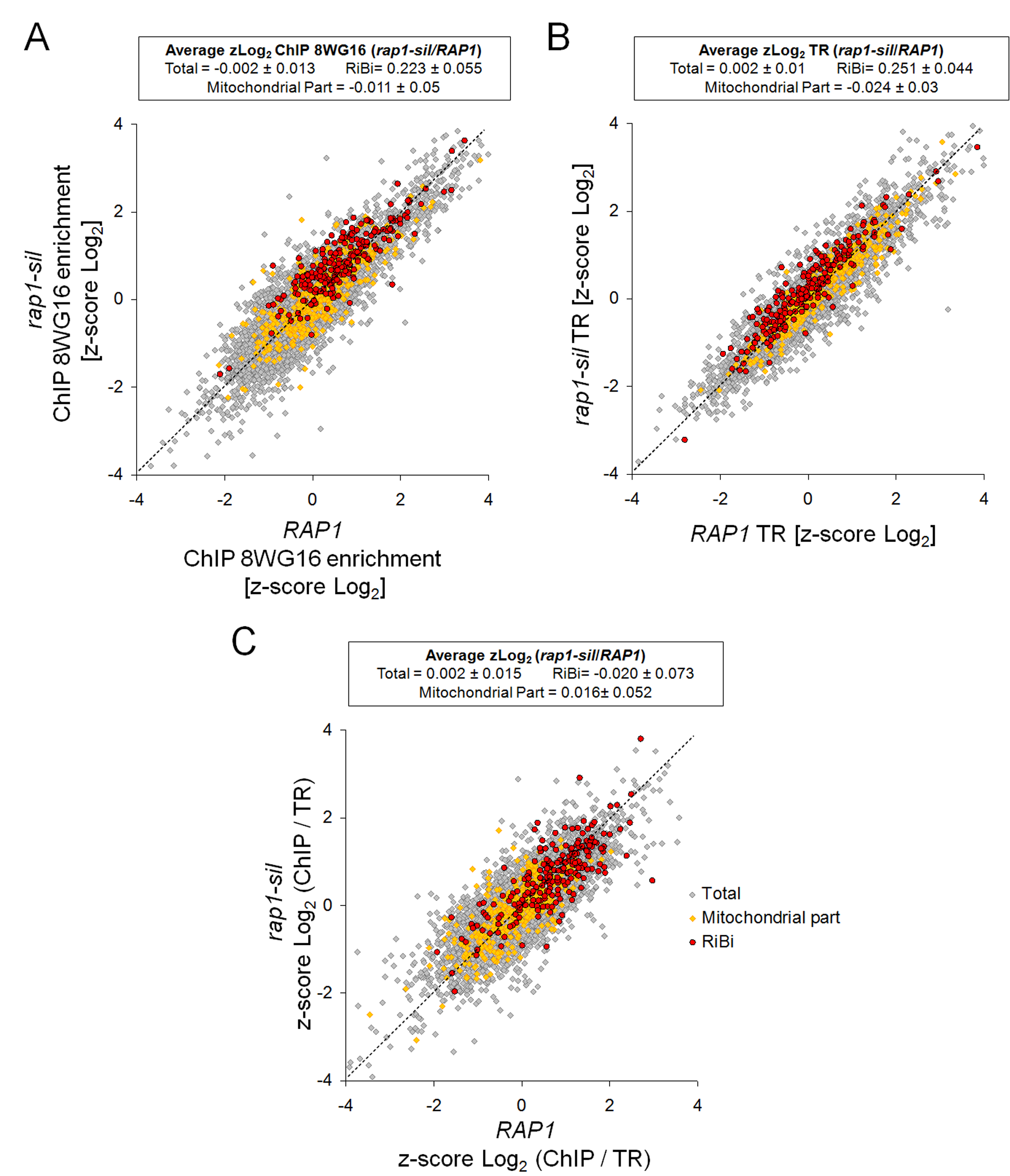

Supplement: Figure S7 — Effect of rap1Δsil on the presence of RNA pol II in the RiBi regulon and mitochondria-related genes. The rap1Δsil mutation slightly increases the RNA pol II levels (A) and transcription rates (B) in the RiBi genes (red dots) without affecting their ChIP/TR ratios (C). No effect on either the RNA pol II levels or transcription rates was detected in mitochondria-related genes (orange diamonds). (1.17 MB TIF) [file pgen.1000614.s007.tif]

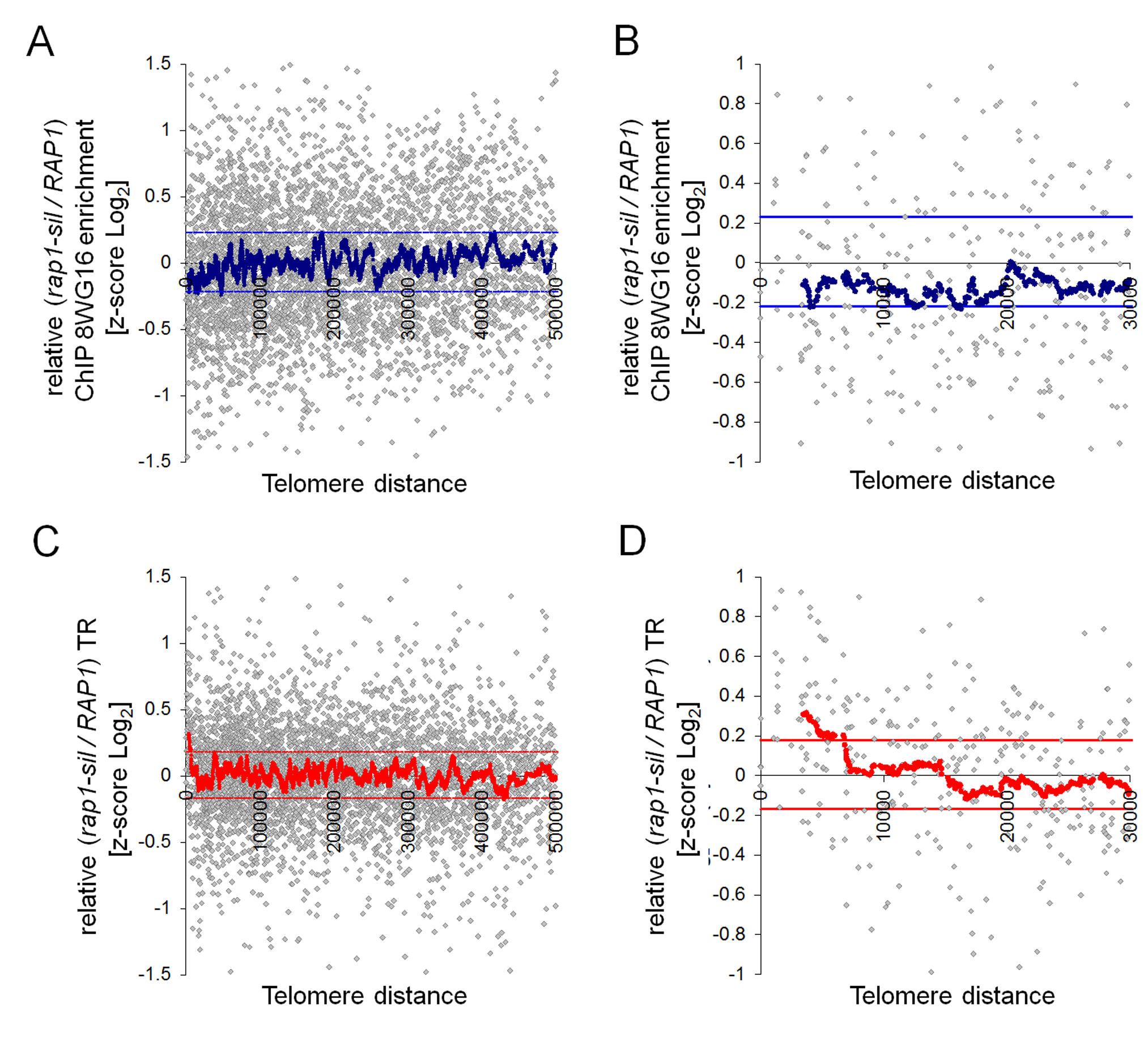

Supplement: Figure S8 — The rap1Δsil mutation increases the proportion of active RNA pol II in subtelomeric genes. The rap1Δsil mutation does not produce significant changes along the length of the chromosome at the total RNA pol II levels, measured by ChIP-on-chip (A, B). It does not produce an increase of active RNA pol molecules along length of the the chromosome length either (C), except within the 10 kb region near the telomeres (extended resolution in D). The red and blue lines represent the averages of the values using a sliding window of 50 genes. Horizontal lines represent the upper and lower limit for this mean using a Shewhart chart with a confidence range of 0.999. (1.22 MB TIF) [file pgen.1000614.s008.tif]

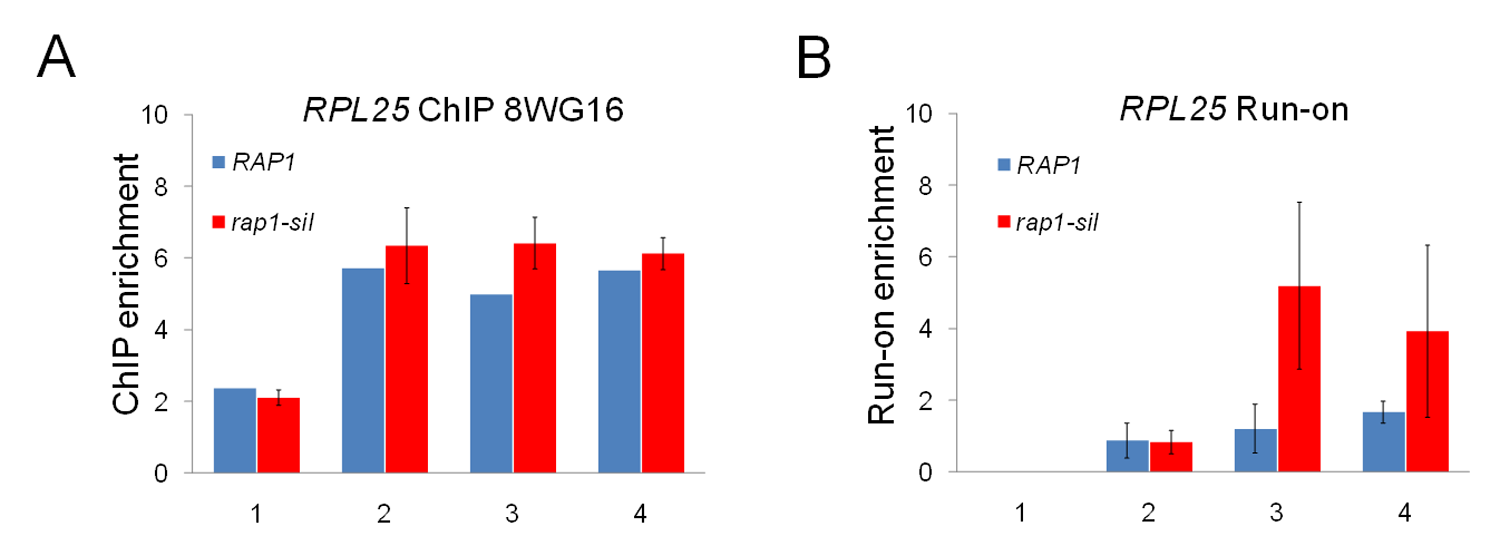

Supplement: Figure S9 — Effect of rap1Δsil on the presence and activity of RNA pol II in RPL25. There is no significant difference in the distribution of RNA pol II molecules within the RPL25 gene (A), but there is a significant increase of active RNA pol II molecules toward the 3′ end of the gene in the rap1Δsil mutant with regard to the wild type RAP1 (B). (0.10 MB TIF) [file pgen.1000614.s009.tif]
