# Supplementary material for: Regulon-Specific Control of Transcription Elongation across the Yeast Genome
Source: PLoS Genet. 2009 Aug 21;5(8):e1000614. doi: 10.1371/journal.pgen.1000614 (PMC2721418; doi:10.1371/journal.pgen.1000614)
Supplement: Table S3 — Yeast strains used in this work. (0.04 MB DOC) [file pgen.1000614.s012.doc]

**Supporting Table S3. Yeast strains used in this work**

| **Yeast strain** | **Relevant genotype** | **Reference** |
| --- | --- | --- |
| ARG3 | *MATa, RPB1:Myc18-Kl TRP1*, *TRP1*::*kanMX4*, otherwise isogenic to BY4741 | Vanti et al (2008) |
| BQS252 | *MATa*, *ura3-52*, derived from FY1679 | (García-Martínez et al. 2004) |
| BY4741 | *MATa; his3Δ1; leu2Δ0; met15Δ0; ura3Δ0* | Euroscarf |
| FY1679 | *MATa/MATα*; *ura3-52/ura3-52*; *trp1Δ63/TRP1; leu2Δ1/LEU2; his3Δ200/HIS3; GAL2/GAL2* | Euroscarf |
| SCR101 | *MATα  ade2 his2 leu2 trp1 rap1::UASgal-RPL25-RAP1 (pR415RAP1)* | (Graham et al. 1999) |
| SCR101 *rap1∆sil* | *MATα  ade2 his2 leu2 trp1 rap1::UASgal-RPL25-RAP1 (pR415rap1∆sil)* | (Graham et al. 1999) |
| SJY25 | *MATα  his3∆1 leu2∆0 lys2∆0 ura3∆0* *trp1::kanMX4* *SPT16-myc* | (Jimeno-González et al. 2006) |
| SJY6 | *MATα  his3∆1 leu2∆0 ura3∆0 trp1::kanMX4 spt16::kanMX4 TetO::SPT16* | (Jimeno-González et al. 2006) |
| Y01089 | BY4741, *MATa  his3 leu2 ura3, met15, tpk2::kanMX4* | Euroscarf |

**References**

García-Martínez J, Aranda A, Pérez-Ortín JE (2004) Genomic run-on evaluates transcription rates for all yeast genes and identifies gene regulatory mechanisms. Mol Cell 15(2): 303-313.

Graham IR, Haw RA, Spink KG, Halden KA, Chambers A (1999) *In vivo* analysis of functional regions within yeast Rap1p. Mol Cell Biol 19(11): 7481-7490.

Jimeno-González S, Gómez-Herreros F, Alepuz PM, Chávez S (2006) Gene-specific requirement for FACT during transcription is related to the chromatin organization of the transcribed region. Mol Cell Biol. 26(23): 8710-8721

Vanti M, Gallastegui E, Respaldiza I, Rodríguez-Gil A, Gómez-Herreros F, Jimeno-González S, Jordan A, Chávez S. (2009) Yeast Genetic Analysis Reveals the Involvement of Chromatin Reassembly Factors in Repressing HIV-1 Basal Transcription. PLoS Genetics 5(1):e1000339.
