# Supplementary material for: Regulon-Specific Control of Transcription Elongation across the Yeast Genome
Source: PLoS Genet. 2009 Aug 21;5(8):e1000614. doi: 10.1371/journal.pgen.1000614 (PMC2721418; doi:10.1371/journal.pgen.1000614)
Supplement: Text S1 — Supplementary Materials and Methods. (0.04 MB DOC) [file pgen.1000614.s016.doc]

**Supplementary Materials and Methods**

**Genomic Run-on (GRO)**

GRO was done essentially as described in [1]. Around 6 x 10^8^ yeast cells were used to perform in vivo transcription. After spinning down cells, they were washed twice in 0.5% of N-lauryl sarcosine sodium sulfate (sarkosyl). After the permeabilization step, cells were recovered by low-speed centrifugation and the supernatant was removed. *In vivo* transcription was performed by resuspending cells in 120 µL of 2.5X transcription buffer (50 mM Tris-HCl pH 7.7, 500 mM KCl, 80 mM MgCl2), 16 µL of AGC mix (10 mM each of CTP, ATP and GTP), 6 µL of DTT (0.1 M) and 16 µL of [α^33^P]UTP (3000 Ci/mmol, 10 µCi/µL). The final volume was adjusted to 300 µL with distilled water and the mix was incubated for 5 minutes at 30ºC to allow transcription elongation. The reaction was stopped by adding 1 ml of cold distilled water to the mix. Cells were recovered by centrifugation to remove the non-incorporated radioactive nucleotide.

Total RNA was isolated using the Fast-Prep (Bio101 Inc.) device by resuspending the cells in 500 µL of LETS buffer (0.1 M LiCl, 10 mM EDTA, 0.2 % SDS, 10 mM Tris-HCl pH 7.4), 200 µL glass beads and 500 of water-saturated acid phenol. Contaminants were removed by extraction with chloroform. Labeled RNA was precipitated by adding 0.1 volume of 5 M LiCl and 2.5 volumes of cold ethanol for, at least, 2 h at -20ºC. After centrifugation at maximum speed for 15 min, labeled RNA was washed once with 70 % ethanol and dried. Total extracted RNA was spectrophotometrically quantified. An aliquot was used for specific radioactivity determination in a scintillation counter. All the *in vivo* labeled RNA was used for hybridization (0.35-3.5 x 10^7^ dpm).

Nylon filters were made using PCR-amplified whole ORF sequences as probes [2]. After pre-hybridizing nylon membranes for 1 h in 5X SSC, 5X Denhart's, 0.5% SDS, 100 µg/mL salmon sperm DNA, hybridizations were performed using 3-5 mL of the same solution containing labeled RNA. Hybridizations were conducted during 40-44 h in a roller oven at 65ºC. After hybridization, filters were washed once in 2X SSC, 0.1% SDS for 30 min, and twice in 0.2X SSC, 0.1% SDS for 30 min. Filters were exposed for up to 7 days to a imaging plate (BAS-MP, FujiFilm) which was read at 50 μm resolution in a phosphorimager scanner (FLA-3000, FujiFilm).

**Chromatin immunoprecipitation of RNA pol II (RPCC)**

50 mL of cells were cross-linked by adding formaldehyde to a final concentration of 1% for 15 minutes at room temperature, and the cross-link was quenched by adding glycine to a final concentration of 125 mM. Cell were washed 4 times with 30 mL ice-cold TBS buffer (20mM Tris-HCl, 140 mM NaCl, pH 7.5) and frozen. Cells were thawed on ice and resuspended in 300 µL lysis buffer (50 mM HEPES-KOH pH 7.5, 140 mM NaCl, 1 mM EDTA, 1% Triton X-100, 0.1% sodium deoxycholate, 1 mM PMSF (Phenylmethylsulfonyl fluoride), 1 mM benzamidine and 1 pill of protease inhibitor cocktail (Roche Diagnostics, Mannheim, Germany) dissolved in every 50 ml of buffer). The equivalent of 0.3 ml of frozen glass beads (425-600 mm; Sigma-Aldrich, St Louis, MO, USA) was added to the cellular suspension and cells were lysed at 4°C for 12 minutes of vortexing at maximum power. Lysis buffer (300 µL) was added and the extract was transferred to a new tube. Sonication of DNA was performed to obtain DNA fragments between 200 and 500 bp, with an average size of 350 bp. Chromatin was sonicated on ice-cold water, with 5 pulses of 30 s at High output (200W) in a Bioruptor (Diagenode SA, Liège, Belgium). Cell debris was removed by centrifugation at 12,000 rpm at 4°C for 5 minutes. An aliquot of 10 µL of this whole cell extract (WCE) was kept as positive control.

The antibodies (Ab) used were mouse monoclonal 8GW16 (30 µg; Covance Inc., Berkeley, CA, USA) or Anti-Myc (a gift from Gustav Amerer), and rabbit polyclonal antibodies against Ser5-phosphorylated CTD and Ser2-phosphorylated CTD (David Bentley’s lab). 50 µL of Dynabeads Rat anti-Mouse IgG or Protein G (Dynal Invitrogen Corp., Carlsbad, CA, USA) were washed with 5 mg/ml bovine serum albumin (BSA) in PBS buffer (140 mM NaCl, 2.7 mM KCl, 10 mM Na2HPO4, 1.8 mM KH2PO4, pH 7,4) and incubated with 10 µg of yeast tRNA and the corresponding Ab overnight in agitation at 4°C. We also included non-Ab samples (NA) as negative controls of the immunoprecipitation process.

To eliminate the excess Ab, beads were washed with PBS/BSA and incubated for 1.5-2 hours in agitation to enrich the samples in the DNA fragments specifically cross-linked to the RNA pol II.

Beads were washed twice with lysis buffer, twice with 360 mM NaCl lysis buffer, twice with wash buffer (10 mM Tris-HCl pH 8.0, 250 mM LiCl, 0.5% Nonidet P-40, 0.5% sodium deoxycholate, 1 mM EDTA pH 8.0, 1 mM PMSF, 1 mM benzamidine and 1 pill of protease inhibitor cocktail/25 mL (Roche), and once with TE (10 mM Tris-HCl, pH 8.0, 1 mM EDTA). Two successive washes were performed with 30 µL of elution buffer (50 mM Tris-HCl pH 8.0, 10 mM EDTA, 1% SDS) by incubating for 10 minutes at 65°C each time. The final volume of all the samples (IP, NA and WCE) was raised to 300 µL with TE and treated overnight at 65°C to reverse the cross-linking.

Proteins were degraded by adding 150 µg of proteinase K for 1h 30 min at 37°C. DNA was purified using QIAquick PCR purification columns (Qiagen Inc., Valencia, CA, USA).

RPCC was done using the aforementioned minichips and 5’-3’-macroarrays to determine RNA pol II distributions and IP/TR ratios.

**DNA amplification and array hybridization**

Ligation Mediated PCR [3] was used for DNA amplification. Briefly,

DNA was blunted by T4 phage DNA polymerase in a reaction volume of 124 µL (T4 DNA Pol buffer, 40 µg/µL BSA, 80 µM dNTPs, 0.6 U T4 DNA Polymerase (Roche, Basel, Switzerland). The reaction was allowed to proceed for 20 minutes at 12°C. After phenol/chloroform/isoamylic alcohol extraction, DNA was ethanol-precipitated in the presence of 12 µg of glycogen and was ligated in a final volume of 50 µL with the annealed linkers oJW102 and oJW103 (1.5 µM of each primer). The reaction was carried out overnight at 16°C and ligated DNA was precipitated and resuspended in 25 µL milliQ water. The ligated DNA was dissolved in a final volume of 40 µL (1x DNA pol buffer:, 2 mM MgCl2, 0.25 mM dNTPs, 1.25 µM oligonucleotide oJW102). The reaction was started by incubating for 2 minutes at 55°C, then pausing to add 10 µL of the reaction mix (1x DNA pol buffer, 2 mM MgCl2 and 5 U DNA polymerase from Biotools, Madrid, Spain). The program was resumed for 5 minutes at 72°C, 2 minutes at 95°C and 33 cycles of 30 s at 95°C, 30 s at 55°C and 2 minutes at 72°C. DNA was precipitated overnight and resuspended in 50 µL of milliQ water. A 5 µL DNA aliquot of the LM-PCR was analyzed on a 1.2% agarose gel to check the size (average size of 300-400 bp) and PCR efficiency.

The PCR product was used as a template for the radioactive labeling reaction. The reaction mixture contained 5-15 µL of amplified DNA in 50 µL (1x DNA pol buffer, 2 mM MgCl2, 0.2 mM dATP, dTTP and dGTP, 25 µM dCTP, 1 µM oJW102, 0.8 µCi/µL α-33P-dCTP and 5 U DNA polymerase). The mix was denatured for 5 minutes at 95°C, annealed 5 minutes at 50°C, and amplified for 30 minutes at 72°C. The reaction product was purified through a ProbeQuant G-50 column (Amersham Biosciences, Piscataway, NJ, USA) to remove unincorporated ^33^P-dCTP and oligonucleotides. The macroarrays used and the hybridizations conditions were the same as those described for GRO.

**Image analysis and data normalization**

Both GRO and RPCC experiments were performed in triplicate by swapping the filters in each replicate among the different sampling times. Images were quantified by using the ArrayVision 7.0 software (Imaging Research, Inc.). The signal intensity for each spot was the background subtracted ARM Density (Artifact Removed Median). Only those values that were 1.35 times above the corresponding background were taken as valid measurements. To compare the RPCC data between experiments, the median binding ratio of the 32 rDNA spots were arbitrarily set as background. Reproducibility of the replicates was checked using the ArrayStat software (Imaging Research, Inc.). Normalization between conditions was done using the global median method for RPCC and as described in [1] for GRO experiments. The ratio between immunoprecipitated (IP) and whole cell extract (WCE) in each experiment (or No-Ab and WCE) after normalization was taken as the binding ratio.

In order to compare RPCC data and GRO, the log data were standardized using z-scores (subtracting the mean and dividing by the standard deviation).

**References**

**1. Garcia-Martinez J, Aranda A, Perez-Ortin JE (2004) Genomic run-on evaluates transcription rates for all yeast genes and identifies gene regulatory mechanisms. Mol Cell 15: 303-313.**

**2. Alberola TM, Garcia-Martinez J, Antunez O, Viladevall L, Barcelo A, et al. (2004) A new set of DNA macrochips for the yeast Saccharomyces cerevisiae: features and uses. Int Microbiol 7: 199-206.**

**3. Ren B, Robert F, Wyrick JJ, Aparicio O, Jennings EG, et al. (2000) Genome-wide location and function of DNA binding proteins. Science 290: 2306-2309.**
